# Large-scale brain network dynamics in very preterm children and relationship with socio-emotional outcomes: an exploratory study

**DOI:** 10.1038/s41390-022-02342-y

**Published:** 2022-11-03

**Authors:** Vanessa Siffredi, Maria Chiara Liverani, Lorena G. A. Freitas, D. Tadros, Y. Farouj, Cristina Borradori Tolsa, Dimitri Van De Ville, Petra Susan Hüppi, Russia Ha-Vinh Leuchter

**Affiliations:** 1 Division of Development and Growth, Department of Paediatrics, Gynaecology and Obstetrics, Geneva University Hospitals, Geneva, Switzerland; 2 Institute of Bioengineering, Center for Neuroprosthetics, Ecole Polytechnique Fédérale de Lausanne, Écublens, Switzerland; 3 Department of Radiology and Medical Informatics, Faculty of Medicine, University of Geneva, Geneva, Switzerland; 4 SensoriMotor, Affective and Social Development Laboratory, Faculty of Psychology and Educational Sciences, University of Geneva, Geneva, Switzerland

## Abstract

**Background:**

Children born very preterm (VPT; <32 weeks’ gestation) are at high risk of neurodevelopmental and behavioural difficulties associated with atypical brain maturation, including socio-emotional difficulties. The analysis of large-scale brain network dynamics during rest allows us to investigate brain functional connectivity and its association with behavioural outcomes.

**Methods:**

Dynamic functional connectivity was extracted by using the innovation-driven co-activation patterns framework in VPT and full-term children aged 6–9 to explore changes in spatial organisation, laterality and temporal dynamics of spontaneous large-scale brain activity (VPT, *n* = 28; full-term, *n* = 12). Multivariate analysis was used to explore potential biomarkers for socio-emotional difficulties in VPT children.

**Results:**

The spatial organisation of the 13 retrieved functional networks was comparable across groups. Dynamic features and lateralisation of network brain activity were also comparable for all brain networks. Multivariate analysis unveiled group differences in associations between dynamical functional connectivity parameters with socio-emotional abilities.

**Conclusion:**

In this exploratory study, the group differences observed might reflect reduced degrees of maturation of functional architecture in the VPT group in regard to socio-emotional abilities. Dynamic features of functional connectivity could represent relevant neuroimaging markers and inform on potential mechanisms through which preterm birth leads to neurodevelopmental and behavioural disorders.

**Impact:**

- Spatial organisation of the retrieved resting-state networks was comparable between school-aged very preterm and full-term children.
- Dynamic features and lateralisation of network brain activity were also comparable across groups.
- Multivariate pattern analysis revealed different patterns of association between dynamical functional connectivity parameters and socio-emotional abilities in the very preterm and full-term groups.
- Findings suggest a reduced degree of maturation of the functional architecture in the very preterm group in association with socio-emotional abilities.

## Introduction

Preterm birth occurs during key phases of interrelated neurobiological processes underlying brain development. This seems particularly relevant in infants born very preterm (VPT; <32 weeks’ gestation). As a consequence, VPT children are at high risk of neurodevelopmental and neurobehavioural difficulties associated with atypical brain structural and functional maturation.^1^

Magnetic resonance imaging (MRI) techniques provide powerful, non-invasive tools to delineate aberrant brain development related to prematurity. In particular, resting-state functional MRI (rs-fMRI) has emerged as a promising tool for studying neural networks underlying typical and atypical brain development.^2^ Functional connectivity (FC) derived from rs-fMRI is defined as the temporal dependence of neuronal activity patterns of anatomically separated brain regions.^3,4^ FC between regions in the absence of goal-directed activity and stimulation are used to identify networks with synchronous, spontaneous neuronal activity, termed resting-state networks.^3,4^ The functional relevance of resting-state networks has been inferred not only from their spatial overlap with regions known to underpin sensori-motor and cognitive functions but also from their overlap with known white-matter anatomical pathways.^5–8^

To date, most studies on rs-fMRI in VPT individuals of different ages from newborn to adolescent have used static FC, i.e., the correlation between the activation in different brain regions over the whole scanning time.^9^ Using a seed-based correlation approach—in which few regions of interest (ROI) time series are selected a priori and then correlation with timecourses of each voxel within this given ROI is averaged^7^—studies in VPT individuals at different ages have found altered FC for various brain regions using ROIs such as superior temporal, amygdala, posteromedial and lateral parietal, prefrontal or lateral and superior sensori-motor regions.^10–14^ Similarly, alterations of different resting-state networks have been found in VPT individuals when using whole-brain data-driven approaches, including FC of the salience, default-mode or frontal networks.^15–18^ However, recent studies suggest that static FC measures might be too simplistic to capture the full extent of resting-state activity as they ignore the inherently dynamic nature of fluctuating activity.^9,19,20^ In this perspective, dynamic approaches have been developed with the potential to identify meaningful variations over time in FC between different brain regions.

Among various methods to identify dynamic FC, the innovation-driven co-activation patterns (iCAPs) framework detects moments of significant transient activity to establish large-scale brain networks and their dynamic properties.^20–22^ Using a whole-brain data-driven approach, this framework not only allows us to extract spatial and temporal characteristics of large-scale brain networks but also temporal overlaps between these different networks. Using the iCAPs framework, recent studies conducted in children, adolescents and adults have found a significant association between large-scale brain dynamics and psychiatric symptoms including depression, anxiety and psychotic symptoms.^23,24^

In VPT children, studies show that approximately 25% of them experience behavioural problems.^25,26^ Of particular interest, socio-emotional abilities have been found to have important implications for the forming of peer relationships, adaptive functioning, academic achievement and mental health.^27–29^ Prematurity has been associated with atypical socio-emotional development as early as the first year of life and extends from difficulties in emotional information processing, social understanding, emotion regulation, socialising, peer relationship as well as internalising problems.^28,30–39^ Importantly, deficits in socio-emotional processing and regulation in early life are considered precursors of later psychiatric and mental health problem.^12,40^ In VPT individuals, these difficulties have indeed been found to be long-lasting, with consequences observed in social, occupational and family functioning through adolescence into adulthood;^28,41,42^ as well as increased risk of developing psychiatric disorders as adults, including depression, bipolar affective disorder, anxiety disorder and schizophrenia.^43–45^ In line with these studies, alteration in structural brain architecture has been associated with atypical socio-emotional development in VPT children and adolescents across different ages (for a review, see refs. ^28,46^). Whole-brain white matter and grey matter abnormalities have been associated with poorer emotional regulation and social functioning as well as a higher risk of internalised problem in young VPT.^38,47,48^ Other studies also suggest associations of socio-emotional problems with structural alteration in specific brain regions in VPT individuals. Frontal regions, in particular orbito-frontal areas as well as frontal white-matter connectivity alteration, have been found to be associated with prosocial difficulties, peer problem, emotional moderation (defined as regulatory skills used to moderate the impact of negative emotionality) and internalised problems in VPT children and adolescents.^49–53^ Structural alterations in temporal regions have also been associated with increased anxiety symptoms^54^ and alterations in the fusiform gyrus, a region known to be involved in the recognition of facial emotional expressions and subtle visual cues of social relations, with social immaturity.^55^ Abnormalities in subcortical structures, including the basal ganglia, caudate, cerebellum, hippocampus, have also been associated with reduced prosocial abilities, social adjustment and mental health.^49,51,56,57^ In addition to atypical structural development, functional alteration has also been observed in association with socio-emotional outcomes in VPT individuals. Using static FC, most of these studies used a ROI approach and specifically targeted amygdala-related FC. Findings show an association between atypical amygdala FC with reduced social participation and social functioning, internalising symptoms and emotion moderation.^10,13,58,59^ Using electroencephalogram, increase right frontal activity was associated with internalised symptoms in VPT young adults.^60^

Altogether, brain functions underpinning socio-emotional difficulties in young VPT children have been rarely explored so far, especially using a whole-brain approach. Moreover, to our knowledge, a dynamic FC approach has not been used until now in school-age VPT children and not in association with socio-emotional abilities. Considering previous studies conducted in paediatric population using a dynamic FC approach and the association found with psychiatric symptoms,^23^ such an approach could provide a better understanding of brain functions underpinning socio-emotional difficulties in VPT children and inform on potential brain functional biomarker of socio-emotional difficulties in this population.

In this exploratory study, we first aim to explore dynamic FC in young VPT compared to full-term children aged 6–9. To this end, the iCAPs framework will be applied and spatial organisation, laterality and temporal dynamics of spontaneous large-scale brain activity will be compared between the two groups. Secondly, in VPT children, we aim to explore the potential associations between dynamic FC and socio-emotional abilities by using multivariate analysis.

## Methods and materials

### Participants

A total of 227 VPT children born before 32 gestational weeks between 1 January 2008 and 1 May 2013, in the Neonatal Unit at the Geneva University Hospital (Switzerland) and followed up at the Division of Child Development and Growth, were invited to participate in the “Vis-à-vis interventional study” between January 2017 and July 2019. VPT children were excluded if they had a Fluid-Crystallized Index (FCI, as measured by the Kaufman Assessment Battery for Children – 2nd Edition (K-ABC-II^61^)) below 70, sensory or physical disabilities (cerebral palsy, blindness, hearing loss), or an insufficient understanding of French.

A total of 45 VPT participants aged between 6 and 9 years were enrolled. Moreover, 17 term-born controls aged between 6 and 9 years were recruited through the community. Of the 62 enrolled participants, 9 participants were excluded as they did not complete both the brain MRI scan and the neuropsychological assessment (VPT, *n* = 7; full-term controls, *n* = 2); and 13 participants were excluded due to high level of motion artefacts in the rs-fMRI sequence (VPT, *n* = 10; full-term controls, *n* = 3). The final sample included 40 participants between 6 and 9 years of age: 28 VPT (15 females, 13 males; mean age in months (standard deviation) = 97.82 (13.92)) and 12 full-term participants (5 females, 7 males; mean age in months (standard deviation) = 94.83 (14.44)) (Table 1).

**Table 1:** Neonatal and demographic characteristics, as well as socio-emotional outcomes of the VPT and full-term participants.

	VPT (*n* = 28)	Full-term (*n* = 12)	Group comparison
Neonatal and demographic characteristics			
Gestational age, mean (SD) in weeks	30.03 (1.58)	39.94 (1.12)	*t* (29.06) = –22.54, ***p*** < **0.001**
Birth weight, mean (SD) in g	1340.00 (353.42)	3464.17 (319.87)	*t* (22.96) = –18.64, ***p*** < **0.001**
Age at assessment, mean (SD) in months	97.82 (13.92)	94.83 (14.44)	*t* (20.61) = 0.55, *p* = 0.606
Sex, *n*	15 females 13 males	5 females 7 males	*X*^2^ (1, *N* = 40) = 0.48, *p* = 0.49
Socio-economic risk, mean (SD)	4.70 (2.35)	2.58 (1.08)	*t* (36.88) = 3.86, ***p*** < **0.001**
Fluid-Crystallized Index (FCI), mean (SD)	106.78 (13.79)	110.75 (10.93)	*t* (26.47) = –0.96, *p* = 0.344
Socio-emotional abilities			
Affect recognition, mean (SD)	–0.18 (0.97)	0.40 (1.06)	*F* (2,36) = 2.156, *p* = 0.131
Theory of mind, mean (SD)	–0.10 (1.01)	0.23 (1.05)	*F* (2,36) = 1.046, *p* = 0.362
Internalised problems, mean (SD)	0.13 (0.95)	–0.3 (1.13)	*F* (2,36) = 0.756, *p* = 0.477
Emotional control, mean (SD)	51.29 (12.19)	49.40 (7.79)	*F* (2,34) = 0.536, *p* = 0.590

Neonatal and demographic characteristics: independent-sample *t*-test or *χ*^2^, as appropriate, were used to compare the VPT and the full-term control groups. Socio-economic status of the parents was estimated using the Largo scale, a validated 12-point score based on maternal education and paternal occupation.^71^ Higher Largo scores reflect lower socio-economic status.

Socio-emotional abilities: for affect recognition, theory of mind and internalised problems, raw scores were regressed on age at testing and the standardised residuals were used as scores. For emotional control, standardised *T*-scores were used (mean = 50, SD = 10). Linear regression model was used to compare the VPT and the full-term groups adding socio-economic status as a covariate in the model.

Significant *p* values are indicated in bold (*p* < 0.05).

All neuropsychological assessments and MRI acquisitions were completed at the Campus Biotech in Geneva, Switzerland. This study was approved by the Swiss Ethics Committees on research involving humans, ID: 2015-00175. Written informed consent was obtained from the principal caregiver and from the participant.

### Demographic and neuropsychological measures

Socio-economic status (SES) of the parents was estimated using the Largo scale, a validated 12-point score based on maternal education and paternal occupation.^62^ Higher Largo scores reflect lower SES of the parents. Hand preference (right or non-right handedness) was established by asking the child which hand he/she uses to write and draw. The K-ABC-II^61^ was used to evaluate the FCI as a measure of general intellectual functioning. The FCI have a mean of 100 and a standard deviation of 15.

Participants’ socio-emotional outcomes were assessed using four different measures:

- Theory of Mind subtest of the Developmental Neuropsychological Assessment – 2nd Edition (NEPSY-II^63^): raw scores were regressed on age at testing and SES (Largo score). Standardised residuals were used as a score, called theory of mind.
- Affect Recognition subtest of the NEPSY-II:^63^ raw scores were regressed on age at testing and SES. Standardised residuals were used as a score, called affect recognition.
- Internalised Score subscale of the Strength and Difficulties Questionnaire – parent version (SDQ):^64,65^ raw scores were regressed on age at testing and SES. Standardised residuals were used as a score, called “internalised problems”. High internalised scores reflect increased internalised difficulties in daily life.
- Emotional Control Scale of the Behaviour Rating Inventory of Executive Function, parent version (BRIEF^66^): standardised scores were used (mean = 50, SD = 10) and regressed on SES. Standardised residuals were used as a score, called emotional control. Higher emotional control scores reflect increased difficulties in emotional control.

Further details are given in Supplementary Methods.

### Magnetic resonance imaging (MRI) acquisition and preprocessing

#### Magnetic resonance imaging acquisition

MRI data were acquired at the Campus Biotech in Geneva, Switzerland, using a Siemens 3T Magnetom Prisma scanner. Structural T1-weighted MP-RAGE (magnetisation-prepared rapid gradient-echo) sequences, resting-state functional images were T2*-weighted with a multislice gradient-echo-planar imaging a fieldmap sequences were acquired (further details in Supplementary Methods).

#### Resting-state functional MRI data preprocessing

Our data were preprocessed using SPM12 (Wellcome Department of Imaging Neuroscience, UCL, UK) in MATLAB R2016a (The MathWorks, Inc., Natick, Massachusetts, United States) and the preprocessing pipeline described by Freitas et al.^67^ (detailed preprocessing in Supplementary Methods).

### Innovation-driven co-activation patterns (iCAPs) and extraction of iCAPs activation measures

#### iCAPs

iCAPs analysis is a novel state-of-the-art rs-fMRI analysis tool that allows us to derive a set of whole-brain spatial patterns of regions whose activity simultaneously increases or decreases, thus characterised by similar functional dynamic behaviour.^22^ For a comprehensive explanation of the methodology and procedure, we refer to Karahanoglu and Van De Ville (2015) and to Zöller et al. (2019). We tailored the openly available MATLAB code (https://c4science.ch/source/iCAPs/) MATLAB vR2016a (The MathWorks, Inc., Natick, MA) to apply the iCAPs framework in VPT and full-term participants. The overall routine is composed of four steps: total activation (TA), detection of significant transients, aggregation, temporal clustering and time course extraction as described in detail in Supplementary Methods.

#### iCAPs labelling

To guide iCAPs labelling, previous studies using the iCAPs framework were used as reference.^23,24,68,69^ Moreover, the Dice coefficient of similarity was completed to examine similarities between the iCAPs retrieved and Yeo’s 17 cortical resting-state networks (further details in Supplementary Methods).^70^

#### Extraction of temporal properties

For computation of temporal properties, iCAPs’ time series were recovered by backprojecting each iCAP into subjects’ activity-inducing signals; i.e., block-type activity representations recovered by TA. For each iCAP, we then computed two measures representing the temporal characterisation of iCAPs: (1) occurrence: the number of activation blocks; (2) total duration: the total duration of overall activation as a percentage of the total non-motion scanning time.

To explore the dynamic interactions during resting-state, coupling and anticoupling duration of each pair of iCAPs were calculated as time points of same-signed or oppositely signed co-activation measured as a percentage of the total non-motion scanning time or as Jaccard score: per cent joint activation time of the two respective iCAPs.

#### Extraction of laterality measure

To explore the laterality of brain networks, the iCAPs maps were co-registered MNI symmetrical template, available at http://www.bic.mni.mcgill.ca/ServicesAtlases/ICBM152NLin2009. Based on previous studies, the laterality of activity maps aimed at exploring possible asymmetry effect between the two hemispheres by comparing lateralised amplitude maps of the iCAPs.^71^ Therefore, the amplitudes of these patterns reflect the mean activity amplitude for each voxel when contributing to a certain network. In order to obtain a Laterality Index (LI) for each voxel, we flipped the left hemisphere maps and subtracted them from unflipped right hemisphere maps.^69^ Positive and negative values in these LI maps reflect, respectively, right and left lateralisation. These maps were then averaged for each iCAPs in order to obtain one LI for each iCAPs and each participant.

### Statistical analyses

#### Group comparisons of iCAPs activation measures

Measures of occurrence and total duration of each iCAPs as well as coupling and anticoupling between each pair of iCAPs were compared between the VPT and full-term groups using a general linear model and adding SES and sex as covariates to the model. The LI measure was compared between the VPT and full-term groups using a general linear model and adding hand preference, SES and sex as covariates to the model. The *p* values were corrected for multiple comparisons with the false discovery rate.^72^ Analyses were performed using the R software version 4.0.3, and R studio version 1.3.1093.^73,74^

#### Multivariate correlation between iCAPs’ timecourses and socio-emotional measures

To evaluate multivariate patterns of correlation between iCAPs temporal characteristic and socio-emotional measures, we used partial least squares correlation (PLSC). A publicly available PLSC implementation in MATLAB was used (https://github.com/danizoeller/myPLS).^75,76^ PLSC is a data-driven multivariate technique that maximises the covariance between two matrices by identifying latent components that are linear combinations of the two matrices, i.e., socio-emotional and iCAPs temporal characteristics measures (detailed procedure is given in Supplementary Methods).^77^

## Results

### Participant characteristics

Of the 62 participants enrolled, 9 participants were excluded as they did not complete both the brain MRI scan and the neuropsychological assessment (VPT, *n* = 7; FT, *n* = 2); and 13 participants were excluded due to high level of motion artefacts in the rs-fMRI sequence described in the previous section (VPT, *n* = 10; FT, *n* = 3). The final sample included 28 VPT and 12 full-term participants between 6 and 9 years of age (Table 1). VPT and full-term participants were comparable for sex, age at assessment and FCI score, as well as for all socio-emotional outcomes. SES of the parents showed a significant group difference, with lower SES (higher Largo score) in the VPT group compared to the full-term group.

### Extracted innovation-driven co-activation patterns (iCAPs)

**Fig. 1:**
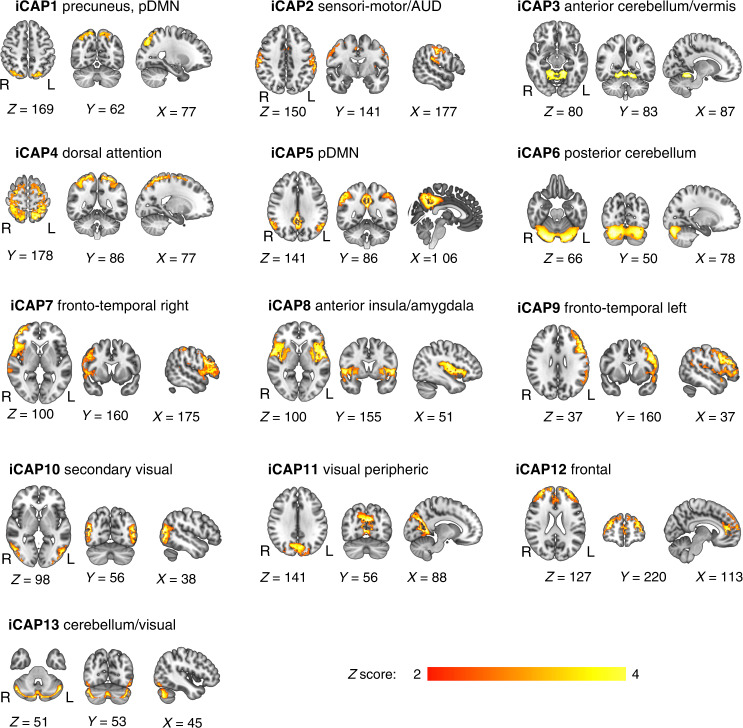
Spatial patterns of the 13 innovation-driven co-activation patterns (iCAPs) retrieved from all participants. These spatial patterns include precuneus/posterior default-mode network (DMN), sensori-motor/auditory (AUD), anterior cerebellum/vermis, dorsal attention, posterior default-mode network (pDMN), posterior cerebellum, fronto-temporal right, anterior insula/amygdala, fronto-temporal left, secondary visual, visual peripheric, frontal, cerebellum/visual. Locations denote displayed slices in Montreal Neurological Institute coordinates.

The iCAPs framework was applied to rs-fMRI scans of both VPT and full-term participants. The 13 extracted spatial maps represented in each iCAP correspond to well-known resting-state networks and were reminiscent of common task-related and cognitive networks typically observed in fMRI studies.^4^ They also correspond to iCAP networks identified in previous studies.^23,24,68,69^ Specifically, the obtained networks included sensory-related networks, i.e., sensori-motor/auditory, visual peripheric and secondary visual; higher-level cognitive network, i.e., the dorsal attention, frontal and fronto-temporal left and right networks; and default-mode network (DMN) decomposed into precuneus/posterior DMN and posterior DMN. The remaining iCAPs comprised anterior insula/amygdala and cerebellar network decomposed into anterior cerebellum/vermis, posterior cerebellum and cerebellum/visual networks (Fig. 1). Supplementary Table S1 shows in detail the iCAPs networks of regions from the automated anatomical labelling 2 atlas (AAL2), and Supplementary Table S1.a shows results from the Dice analyses.

### Group comparison of temporal properties of networks

We compared the VPT and the full-term groups for each temporal property considered: the occurrence and the total duration for each iCAPs, as well as the per cent joint activation time of coupling and anticoupling for each pair of iCAPs. There was no significant group difference for any of the temporal measures after multiple comparison corrections (Supplementary Tables S2–S5).

### Group comparison of laterality of networks

We compared the VPT and the full-term groups for the LI measure. There was no significant group difference for the laterality measure after multiple comparison corrections (Supplementary Table S6).

### Association between iCAPs’ dynamics and socio-emotional measures

**Fig. 2:**
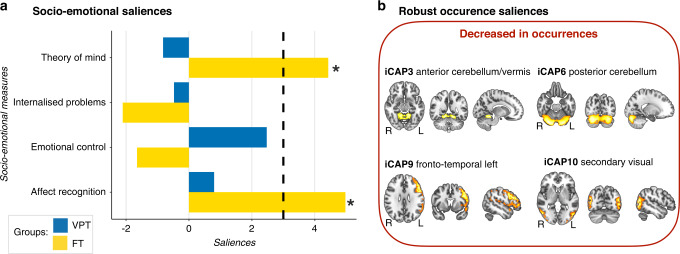
Associations between socio-emotional abilities and occurrence of the 13 iCAPs in the very preterm (VPT) and full-term (FT) groups based on the PLSC analysis. **a** The diverging graph shows bootstrap ratio *z*-scores (*x*-axis) for each socio-emotional measures (*y*-axis) for the VPT and the FT groups in blue and yellow, respectively. Socio-emotional measures with an absolute bootstrap ratio *z*-score ≥3 or ≤–3 (indicated by a back dash-dotted line on the graph) yield a robust contribution to the component (indicated by a black star). **b** Only robust bootstrap ratio *z*-scores (absolute bootstrap ratio *z*-scores above or equal to 3) for iCAPs’ occurrences are shown.

First, the PLSC analysis applied to socio-emotional scores and occurrence of the 13 iCAPs in the VPT and the full-term groups identified one statistically significant latent components: latent component 1 (*p* = 0.006). Latent component 1 revealed a pattern of significant association in the full-term group only (Fig. 2). In full-term children, increased socio-emotional abilities (i.e., affect recognition and theory of mind) were associated with increased occurrence of iCAP3-anterior cerebellum/vermis, iCAP6-posterior cerebellum, iCAP9-fronto-temporal left, iCAP10-visual secondary. In the VPT group, there was no significant association between socio-emotional abilities and occurrences of the 13 iCAPs network.

Second, the PLSC analysis applied to socio-emotional measures and the total duration of the 13 iCAPs in the VPT and the full-term groups show no significant latent component.

**Fig. 3:**
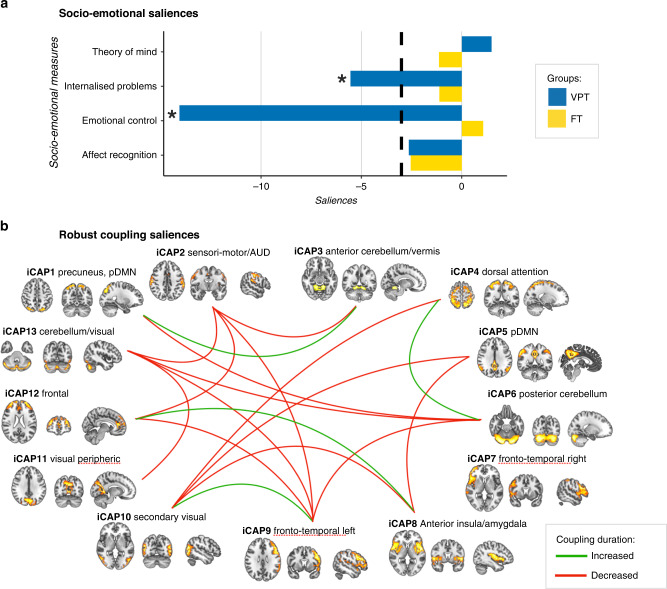
Associations between socio-emotional abilities and coupling of the 13 pairs of iCAPs in the very preterm (VPT) and full-term (FT) groups based on the PLSC analysis. **a** The diverging graph shows bootstrap ratio *z*-scores (*x*-axis) for each socio-emotion measure (*y*-axis) for the VPT and the FT groups in blue and yellow, respectively. Socio-emotional measures with an absolute bootstrap ratio *z*-score ≥3 or ≤–3 (indicated by a back dash-dotted line on the graph) yield a robust contribution to the component (indicated by a black star). **b** Only robust bootstrap ratio *z*-scores (absolute bootstrap ratio *z*-scores ≥3 or ≤–3) for iCAPs’ couplings are shown.

Third, the PLSC analysis applied to socio-emotional measures and coupling duration of the 13 iCAPs in the VPT and the full-term groups identified one statistically significant latent components: latent component 1 (*p* = 0.002). Latent component 1 revealed a pattern of significant association in the VPT group only (Fig. 3). In VPT children, increased socio-emotional abilities (i.e., decreased difficulties in emotional control and internalised problems) were associated with patterns of increased and decreased duration of coupling between the different iCAP networks. More specifically, in VPT, better socio-emotional abilities were associated with increased coupling duration between iCAPs 8 and 12 (anterior insula/amygdala to frontal network), iCAPs 9 and 10 (fronto-temporal left to secondary visual), iCAPs 4 and 6 (dorsal attention to posterior cerebellum) and iCAPs 1 and 3 (precuneus, pDMN to anterior cerebellum/vermis); and with decreased coupling duration between iCAPs 5 and 8 (posterior DMN to anterior insula/amygdala), iCAPs 1 and 6 (precuneus, pDMN to posterior cerebellum), iCAPs 6 and 9 (posterior cerebellum to fronto-temporal left), iCAPs 2 and 9 (sensori-motor/auditory to fronto-temporal left), iCAPs 9 and 13 (fronto-temporal left to cerebellum/visual), iCAPs 11 and 13 (visual peripheric to cerebellum/visual), iCAPs 5 and 10 (posterior DMN to secondary visual), iCAPs 4 and 10 (dorsal attention to secondary visual), iCAPs 6 and 13 (posterior cerebellum to cerebellum/visual), iCAPs 9 and 12 (fronto-temporal left to frontal), iCAPs 2 and 13 (sensori-motor/auditory to cerebellum/visual), iCAPs 2 and 3 (sensori-motor/auditory to anterior cerebellum/vermis), iCAPs 8 and 10 (anterior insula/amygdala to secondary visual), iCAPs 2 and 12 (sensori-motor/auditory to frontal).

Finally, the PLSC analysis applied to socio-emotional measures and anticoupling duration of the 13 iCAPs in the VPT and the full-term groups show no significant latent component.

Original saliences as well as their bootstrap-estimated standard deviations and bootstrap ratio *z*-scores for the PLSC analyses showing a significant latent component are reported in Supplementary Table S8.

## Discussion

Using a novel whole-brain data-driven approach tailored to disentangle transient activity of coordinated brain networks from spontaneous rs-fMRI measurements, we unravelled large-scale resting-state dynamics in VPT and full-term children aged 6 to 9 years. In this cohort of well-functioning VPT children, spatial organisation of the 13 networks retrieved was comparable to full-term controls. Dynamic features and lateralisation of network brain activity were also comparable across groups for the 13 large-scale brain networks. Despite apparent similarities in terms of dynamical FC parameters, multivariate analysis unveiled group differences in their associations with socio-emotional abilities. While a pattern of decreased engagement in certain brain networks was associated with better socio-emotional abilities in full-term controls; in the VPT group, better socio-emotional abilities were associated with coordination of activity across different networks, i.e., coupling duration between different pairs of networks.

In this cohort of well-functioning VPT children, overall dynamic FC characteristics were comparable to the full-term control group. First, spatial organisation of spontaneous brain activity was similar across the two groups. Second, temporal dynamics of iCAPs brain network were comparable in terms of occurrence and duration of engagement in a given network as well as in terms of coupling and anticoupling between pairs of networks. Moreover, the lateralisation of the large-scale brain networks retrieved was also comparable across groups. This pattern of dynamic FC similarities between VPT and full-term children is not consistent with previous studies using static FC.^14,78^ These studies found significant group differences across VPT and full-term school-age children with differences in terms of connectivity strength of different resting-state networks, e.g., DMN, sensori-motor network and the visual and dorsal attention network. Nevertheless, the measure of connectivity strength used in the context of static FC might be complementary but not comparable to the dynamic measures used in the context of dynamic FC where we explore the occurrences, the duration and the joint activation time of different networks. To the best of our knowledge, there is currently no study in school-age preterm children exploring dynamic FC. In preterm and full-term infants, Stoecklein et al. (2020) recently used FC variability or moment-to-moment variations of BOLD signals that represent neural dynamic range.^79^ Whole-brain FC variability was highly similar between 50 preterm and 25 full-term infants (for which the authors replicated the variability map in publicly available data of healthy term-born infants of the Developmental Human Connectome Project) at term equivalent age suggesting that prematurity does not influence significantly FC variability at term equivalent age. Another explanation for the discrepancies with previous static FC studies showing brain functional differences between VPT and full-term children is that, in the current study, children with general intellectual abilities below 70, as well as children with high rates of motion artefact in rs-fMRI time series, were excluded. In our study, the FCI was comparable for the two groups and within the average range. It is therefore possible that only well-functioning children with good self-regulation abilities were included in the analyses. A recent study by Bolton et al. (2020) specifically examined motion characteristics during rs-fMRI acquisition in healthy adults.^80^ Results show a broad array of behavioural and clinical characteristics related to motion including, among other, self-regulation, anxiety and depression. During childhood, it is possible that the association between motion and behavioural characteristics is exacerbated leading to the exclusion of children with increased behavioural difficulties in everyday life and thus greater atypicality in functional brain organisation.

The iCAPs framework has previously been found to provide relevant makers of psychiatric symptomatology in adults and children, such as depression, anxiety and psychotic symptoms.^23,24^ In the current study, despite comparable dynamic features of resting-state networks between VPT and full-term controls, different patterns of association with socio-emotional outcomes were observed across the two groups. In full-term controls, increased socio-emotional abilities were associated with a reduction in the occurrence of various resting-state networks, including the anterior cerebellum/vermis, posterior cerebellum, fronto-temporal left, and visual secondary networks. On the opposite, in VPT children, socio-emotional abilities were associated with a pattern of increased and decreased coupling between networks. These results are consistent with previous studies suggesting that in VPT individuals' cognitive abilities are more dependent on coupling between networks than in full-term controls, for whom cognitive abilities are more related to the involvement of a given network.^81–84^ Altogether, the findings suggest that, while in the full-term control group socio-emotional abilities were associated with different degrees of integration of functional brain networks, i.e., recruitment of intra-network FC; in the VPT group, socio-emotional abilities were associated with a different degree of segregation between networks, i.e., increase and decrease in recruitment of inter-network FC. According to previous studies, typical development of functional architecture appears to show ongoing changes with an increase in integration along with a decrease in segregation of functional networks as children get older.^85–87^ Structurally this increased segregation and loss of increased integration has been shown at different ages in preterm compared to full-term infants and children.^88,89^ It is therefore possible that the group difference observed in the pattern of association between dynamic FC features and socio-emotional abilities reflect a reduced degree of maturation of the functional architecture in the VPT group with the recruitment of different degree of segregation between FC networks, instead of different degree of integration as found in the full-term control group. These results are consistent with the study of Muñoz-Moreno et al. (2016)^90^ showing that socio-emotional scores were related to structural segregation metrics (local efficiency and clustering) between 1- and 2-yearold. In this context, longitudinal data could inform on the developmental trajectories and maturation of dynamic FC and its association with socio-emotional abilities in VPT children.

While providing new insights into the dynamic functional organisation in children born VPT, the current study has a number of limitations that need to be considered. First, the sample size of the study is rather small and warrants validation in larger samples. The small sample size may have limited the identification of subtle group differences in FC dynamics. Compared to previous studies using dynamic FC in different clinical populations,^23,24,68,79^ a minimum of 20–30 participants per group included in the dynamic FC analyses could be considered an ideal sample size. In addition to a small sample size overall, we were not able to recruit an equal number of participants in the two groups (28 VPT and 12 full-term). Families of control participants within the current age range (6–9-year-old) were difficult to find and motivate for an MRI study. Moreover, the study included only intellectually well-functioning VPT children. The exclusion of 13 participants (i.e., 20% of the cohort) due to significant head motion during rs-fMRI acquisition might have accentuated this point even more through the exclusion of children with increased behavioural difficulties. In regards to the high percentage of motion in our sample, the use of a movie during the resting-state session, such as recently published by Vanderwal and colleagues (2015),^91^ might help reduce motion during this sequence in young children. This movie, called *Inscapes*, features abstract shapes without a narrative or scene-cuts and was designed to provide enough stimulation to improve compliance related to motion and wakefulness while minimising cognitive load during the collection of resting-state functional MR images. Moreover, considering the role of hand preference in the establishment of lateralisation of network,^92^ the use of a fine measure of hand preference, e.g., Edinburgh handedness inventory, would be more appropriate than the one currently used in the context of this study. Finally, social environment has been found to have a significant impact on socio-emotional development.^28^ In the current study, the impact of SES, as measured by the Largo scale, was considered and controlled in our analyses. Nevertheless, further environmental influences might contribute significantly, such as family functioning, parental mental health and parenting strategies as well as peer relationship. Taking these limitations into account, the dynamic features of FC could represent relevant neuroimaging markers and inform on potential mechanisms through which preterm birth leads to neurodevelopmental deficits. In this context, longitudinal data could inform on the developmental trajectories and maturation of dynamic FC and its association with socio-emotional abilities in VPT children.

## Conclusions

Leveraging recent advances in the analysis of dynamic features of FC, we explored precise moments of brain network activation and interaction in VPT children compared to full-term children from 6 to 9 years of age. Despite group similarities in terms of dynamical FC parameters, multivariate analysis revealed a different pattern of association with socio-emotional abilities in the VPT and full-term groups, which might reflect a reduced degree of maturation of the functional architecture in the VPT group.

## Supplementary information

SupplementaryMaterials_DynamicFCinVPT

## Data Availability

Deidentified individual participant data (including data dictionaries) will be made available, in addition to study protocols, the statistical analysis plan, and the informed consent form. The data will be made available upon publication to researchers who provide a methodologically sound proposal for use in achieving the goals of the approved proposal. Proposals should be submitted to Russia.HaVinhLeuchter@unige.ch.
