# Supplementary material for: Large-scale brain network dynamics in very preterm children and relationship with socio-emotional outcomes: an exploratory study
Source: Pediatr Res. 2022 Nov 3;93(7):2072–80. doi: 10.1038/s41390-022-02342-y (PMC10313511; doi:10.1038/s41390-022-02342-y)
Supplement: Supplementary file 1 — SupplementaryMaterials_DynamicFCinVPT [file 41390_2022_2342_MOESM1_ESM.pdf]

Supplementary Materials

Supplementary Methods

Participants

227 VPT children born before 32 gestational weeks between 01.01.2008 and 01.05.2013, in the Neonatal Unit at the Geneva University Hospital (Switzerland) and followed up at the Division of Child Development and Growth, were invited to participate in the "Vis-à-vis interventional study" between January 2017 and July 2019. VPT children were excluded if they had a Fluid-Crystallized Index (FCI, as measured by the Kaufman Assessment Battery for Children – 2nd Edition (K-ABC-II [63])) below 70, sensory or physical disabilities (cerebral palsy, blindness, hearing loss), or an insufficient understanding of French. These exclusion criteria were determined based on medical record hold by the Division of Child Development and Growth at the Geneva University Hospital (Switzerland). The FCI was also completed as part of the current study (see Methods section). A total of 45 VPT participants aged between 6 to 9-year-old were enrolled. Of note, we attribute the small percentage of participation in the current study, i.e., 19.8 %, due to the fact that the data collected here were part of an intervention study mentioned above "Vis-à-vis interventional study". The data used in the current study are the data collected at baseline, before any intervention were conducted. Moreover, 17 term-born controls aged between 6 to 9-year-old were recruited through the community.

Of the 62 enrolled participants, 9 participants were excluded as they did not complete both the brain MRI scan and the neuropsychological assessment (VPT, n=7; full-term controls, n=2); and 13 participants were excluded due to high level of motion artefacts in the rs-fMRI sequence (VPT, n=10; full-term controls, n=3). The final sample included 40 participants between 6 and 9 years of age: 28 VPT (15 females, 13 males; mean age in months (standard deviation) = 97.82 (13.92)) and 12 full-term participants (5 females, 7 males; mean age in months (standard deviation) = 94.83 (14.44)), Table 1.

Demographic and Neuropsychological measures

Socio-economic status of the parents was estimated using the Largo scale, a validated 12-point score based on maternal education and paternal occupation.[64] Higher largo scores reflect lower socio-economic status of the parents. This score was developed by R Largo developmental paediatrician for large population based outcome studies and particularly adapted to the Swiss population.

Hand preference (i.e., right or non-right handedness) was established by asking the child which hand he/she uses to write and draw.

The Kaufman Assessment Battery for Children – 2nd Edition (K-ABC-II [63]) was used to evaluate the Fluid-Crystallized Index (FCI) as a measure of general intellectual functioning. For children younger than 7 years of age (i.e., in the VPT group, n= 7; in the FT group, n=3), the FCI is

derived from a linear combination of 11 core subtests; and for older children, it is derived from 10 core subtests. The FCI have a mean of 100 and a standard deviation of 15.

Participants' socio-emotional outcomes was assessed using four different measures:

- The Theory of Mind subtest of the NEPSY-II [65] giving a total score measuring the ability to understand mental contents, such as belief, intention or deception. As the Theory of Mind subtest does not provide a standard score, raw scores were regressed on age at testing and socio-economic status (i.e., Largo score). Standardised residuals were used as a score, called theory of mind.
- The Affect Recognition subtest of the Developmental Neuropsychological Assessment - 2nd Edition (NEPSY-II [65]) giving a total score assessing facial emotional recognition. For consistency with the previous NEPSY-II subtest, raw scores were regressed on age at testing and socio-economic status (i.e., Largo score). Standardised residuals were used as a score, called affect recognition.
- The Internalised Score subscale of the Strength and Difficulties Questionnaire – parent version (SDQ), was used to assess emotional and peer problems in daily life.[66,67] It assesses participant's internalised difficulties over the previous 6 months. The Internalised Score of the SDQ is scored on a Likert scale and is the sum of the scales emotional and peer problems. As standardised scores are not available for this measure, raw scores were regressed on age at testing and socio-economic status (i.e., Largo score). Standardised residuals were used as a score, called" internalised problems". High internalised scores reflect increased internalised difficulties in daily life.

- The Emotional Control Scale of the Behaviour Rating Inventory of Executive Function, parent version (BRIEF [68]) was used to measure the extent to which the child is able to mediate emotional responses in daily life. As standardised scores are available for this measure, standardised scores were used (mean = 50, SD = 10) and regressed on socio-economic status (i.e., Largo score). Standardised residuals were used as a score, called emotional control. Higher emotional control scores reflect increased difficulties in emotional control.

Magnetic Resonance Imaging (MRI) acquisition and preprocessing

Magnetic Resonance Imaging acquisition

MRI data were acquired at the Campus Biotech in Geneva, Switzerland, using a Siemens 3T Magnetom Prisma scanner. All participants completed a simulated "mock" MRI session prior to their MRI scan. This preparation process was conducted by trained research staff and allowed participants to familiarise themselves with the scanner and the scanning process, eventually raising any concerns they might have had prior to the MRI scan. Furthermore, this process is known to facilitated acquisition of good quality MRI images in children.[69,70] Structural T1-weighted MP-RAGE (magnetization-prepared rapid gradient-echo) sequences was acquired using the following parameters: voxel size = 0.9 × 0.9 × 0.9 mm^3; repetition time (TR) = 2,300 ms; echo time (TE) = 2.32 ms; inversion time (TI) = 900 ms; flip angle (FA) = 8°; and field of view (Fov) = 240 mm. Resting-state functional images were T2*-weighted with a multislice gradient-echo-planar imaging (EPI) sequence

of 64 slices; voxel size = 2 x 2 x 2 mm^3; TR = 720 ms; TE = 33 ms; Fov = 208 mm; 500 frames; acquisition time = 6 minutes. During the rs-fMRI sequence, children were asked to keep their eyes closed and engage in mind wandering. These children were constantly monitored and research staff were talking to the children before and after each MRI sequence. Moreover, an eye-tracking camera in the MRI scanner allowed to see the children's eyes and evaluate their awakening level. It is therefore unlikely that any of these children felt asleep during rs- fMRI acquisition. Finally, a fieldmap was acquired each time a participant entered the scanner, with TR = 627 ms; TE1 = 5.19 ms; TE2 = 7.65 ms; and FA = 60°.

Resting-State functional MRI data preprocessing

Our data were preprocessed using SPM12 (Wellcome Department of Imaging Neuroscience, UCL, UK) in MATLAB R2016a (The MathWorks, Inc., Natick, Massachusetts, United States). Rs-fMRI data were converted from the native DICOM to NIFTI format and the preprocessing pipeline described by Freitas, Liverani and colleagues was used.[71] Rs-fMRI images were spatially realigned and unwarped using the fieldmap images acquired, respectively, to correct for motion artefacts and potential geometric distortions. The unwarping step allows to improve the co-registration between structural and functional images and to reduce the distortion variability across subjects during spatial normalisation to a common space.[72] Functional images were then coregistered to their corresponding structural images in subject space and smoothed with a Gaussian filter of full width at half maximum (FWHM) = 6 mm. Structural images were segmented with the SPM12 segmentation algorithm to automatically identify different tissue types within the images, i.e., grey matter, white matter, cerebrospinal fluid;[73] and a study-specific

template was generated using Diffeomorphic Anatomical Registration using Exponential Lie algebra (DARTEL [74]) that will be used in the Innovation-Driven Co-Activation Patterns (iCAPs) framework described below. Finally, the first five rs-fMRI images were excluded, and average white matter and cerebrospinal fluid signals were regressed out from the BOLD time series. For each participant, preprocessed functional images were also check individually for inclusion of the whole cerebrum and of the cerebellum.

Head motion

For rs-fMRI data, the mean framewise displacement for each frame was computed to quantify the extent of head motion from volume to volume for each participant.[75,76] Following Power and colleagues' recommendations, volume censoring ("scrubbing") for motion correction were applied to frames with a mean framewise displacement above 0.5 mm, as well as one frame before and two after. Moreover, if more than 20% of the frames had to be scrubbed, the participant was removed from further analyses. Based on these criteria, 10 VPT and 3 full-term control participants were excluded from further analyses.

Innovation-Driven Co-Activation Patterns (iCAPs) and extraction of iCAPs activation measures

Innovation-Driven Co-Activation Patterns (iCAPs)

Innovation-driven-co-activation-pattern (iCAP) analysis is a novel state-of-the-art rs-fMRI analysis tool that allows to derive a set of whole-brain spatial patterns of regions whose activity simultaneously increases or decreases, thus characterised by similar functional dynamic behaviour.[22] For a comprehensive explanation of the methodology and procedure, we refer to Karahanoglu and colleagues (2015) and to Zöller and colleague (2019). We tailored the openly available MATLAB code (https://c4science.ch/source/iCAPs/) MATLAB vR2016a (The MathWorks, Inc., Natick, MA) to apply the iCAPs framework in VPT and full-term participants. The overall routine is composed of 4 steps:

- Total activation (TA): First, TA applies a voxel-wise hemodynamically-informed deconvolution [77,78] to the fMRI timeseries in a way that promotes the temporal sparsity of activity transients and spatially coherent activations. TA provides three types of information: (1) activity-related signals that are denoised fMRI signals; (2) sustained, or block-type, activity-inducing signals that are deconvolved signals; (3) innovation signals that are the derivative of the activity-inducing signals and encode transient brain activity episodes as spikes.
- Detection of significant transients: Innovation signals are computed as the temporal derivative of the deconvolved signals. The obtained signals can be seen as a representation in terms of transients in brain activity, where large amplitude transients implicitly identify change-points. Significant transients were determined using a two-step thresholding procedure. A temporal threshold estimated from a surrogate distribution, keeping only transients larger than 95% or lower than 5%. Then, a spatial thresholding procedure was applied, in which a frame was considered significant if at least 5% of the grey matter voxels were active. The frames

showing significant transients are called innovation frames, and allow to identify time-points when a given region in the brain undergoes an increase or decrease in activity.

- Aggregation: The significant, i.e. innovation, frames were warped into MNI (Montreal Neurologic Institute) space via a study-specific DARTEL template previously created (see preprocessing). All frames were then aggregated for clustering.
- Temporal clustering: The retained innovation frames, underwent K-means clustering to label timepoints with consistent brain patterns of transitions, i.e., iCAPs. The optimum number of 13 clusters was determined by consensus clustering and following recommendation of previous studies [23,78,79] (Supplementary Figure S1 & S2).
- Time courses extraction: Time courses were obtained for all iCAPs using spatiotemporal transient-informed regression.[21]

iCAPs labelling

To guide iCAPs labelling, previous studies using the iCAPs framework were used as reference. [23,24,80,81] Moreover, the Dice coefficient of similarity was completed to examined similarities between the iCAPs retrieved and the Yeo's 17 cortical resting-state networks.[82] The Dice coefficient is a measure of the extent of overlap between activation maps obtained on two or more occasions.[83,84] It was calculated as follow: $2 \times V_{overlap} / (V_1 + V_2)$

$V_{overlap}$ is the number of overlapping voxels, V_1 is the number of voxels activated in image 1, and V_2 is the number of voxels activated in image 1. A Dice coefficient of 0 implies no overlap at all between activations, whereas a Dice coefficient of 1 implies perfect overlap.

Extraction of temporal properties

For computation of temporal properties, iCAPs' time series were recovered by backprojecting each iCAP into subjects' activity-inducing signals; i.e., block-type activity representations recovered by TA. For each iCAP, we then computed two measures representing the temporal characterisation of iCAPs: 1) occurrence, i.e., the number of activation blocks; 2) total duration, i.e., the total duration of overall activation as percentage of the total non-motion scanning time.

To explore the dynamic interactions during resting-state, coupling and anticoupling duration of each pair of iCAPs were calculated as time points of same-signed or oppositely signed coactivation measured as percentage of the total non-motion scanning time or as Jaccard score; i.e., percent joint activation time of the two respective iCAPs.

Extraction of laterality measure

To explore the laterality of brain networks, the iCAPs maps were co-registered MNI symmetrical template, available at http://www.bic.mni.mcgill.ca/ServicesAtlases/ICBM152NLin2009. Based on previous studies, the laterality of activity maps aimed at exploring possible asymmetry effect between the two hemispheres by comparing lateralised amplitude maps of the iCAPs.[85] Therefore, the amplitudes of these patterns reflect the mean activity amplitude for each voxel when contributing to a certain network. In order to obtain a Laterality Index (LI) for each voxel, we flipped the left hemisphere maps and subtracted them from unflipped right hemisphere maps.[81]

Positive and negative values in these LI maps reflect, respectively, right and left lateralisation. These maps were then averaged for each iCAPs in order to obtain one LI for each iCAPs and each participant.

Statistical analyses

Group comparisons of iCAPs activation measures

Measures of occurrence and total duration of each iCAPs as well as coupling and anticoupling between each pair of iCAPs were compared between the VPT and full-term groups using general linear model and adding socio-economic status and sex as covariate to the model. The LI measure was compared between the VPT and full-term groups using general linear model and adding hand-preference, socio-economic status and sex as covariate to the model. The p values were corrected for multiple comparisons with the false discovery rate (FDR [86]). Analyses were performed using the R software version 4.0.3, and R studio version 1.3.1093.[87,88]

Multivariate correlation between iCAPs' time courses and socio-emotional measures

To evaluate multivariate patterns of correlation between iCAPs temporal characteristic and socio-emotional measures, we used partial least squares correlation (PLSC). A publicly available PLSC implementation in MATLAB was used: https://github.com/danizoeller/myPLS.[89,90] PLSC is a data-driven multivariate technique that maximizes the covariance between two matrices by identifying latent components which are linear combinations of the two matrices, i.e., socio-emotional and iCAPs temporal characteristics measures.[91]

For the socio-emotional scores, the 4 scores of affect recognition, theory of mind, internalised problems and emotional control were considered. For affect recognition, theory of mind and internalised problems, raw scores were regressed on age at testing and socio-economic status (i.e., Largo score) confounds, and the standardised residuals were used for each score. For emotional control, standard scores were regressed on socio-economic status (i.e., Largo score) and the standardised residuals were used. Socio-emotional scores were stored in a 40 x 4 matrix denoted X, in which each row represents one participant and the matrix's 4 columns consist of the 4 socio-emotional scores considered. For each iCAPs temporal characteristics, PLSC was repeated four times, each time considering only one temporal characteristics, i.e., occurrence, total duration, coupling, anticoupling. First, occurrence measures were gathered in a 40 x 13 matrix denoted Y, with each row matching one participant and each column representing the occurrence of each of the 13 iCAPs. Second, total duration measures were gathered in a 40 x 13 matrix denoted Y, with each row matching one participant and each column representing the total duration of each of the 13 iCAPs. Third, coupling measures were stored in a 40 x 78 matrix denoted Y, in which each row represents one participant and the matrix's 78 columns consists of the coupling duration for each pair of iCAPs. Finally, anticoupling measures were stored in a 40 x 78 matrix denoted Y, in which each row represents one participant and the matrix's 78 columns consists of the anticoupling duration for each pair of iCAPs. A cross-covariance matrix was then computed between X (participants x socio-emotional scores) and Y (participants x iCAPs temporal characteristics). Singular value decomposition was then applied to this cross-covariance matrix, resulting in latent components. Statistical significance of multivariate correlation patterns, i.e., latent component, was assessed with permutation testing (1000 permutations) and considered robust at $p<0.01$ following guidelines of previous studies.[23,91] Stability of saliences

were estimated using bootstrapping (500 bootstrap samples with replacement). Bootstrap ratio z-scores for each socio-emotional measure and iCAPs temporal characteristics were obtained by dividing each socio-emotional and iCAPs temporal characteristics weight by its bootstrap-estimated standard deviation, and a p-value was obtained for each bootstrap ratio z-score. The contribution of socio-emotional and iCAPs temporal characteristics weights for a given latent component was considered robust at $p < 0.01$ (i.e., absolute bootstrap ratio z-scores above 3 or below -3).

Supplementary Figures

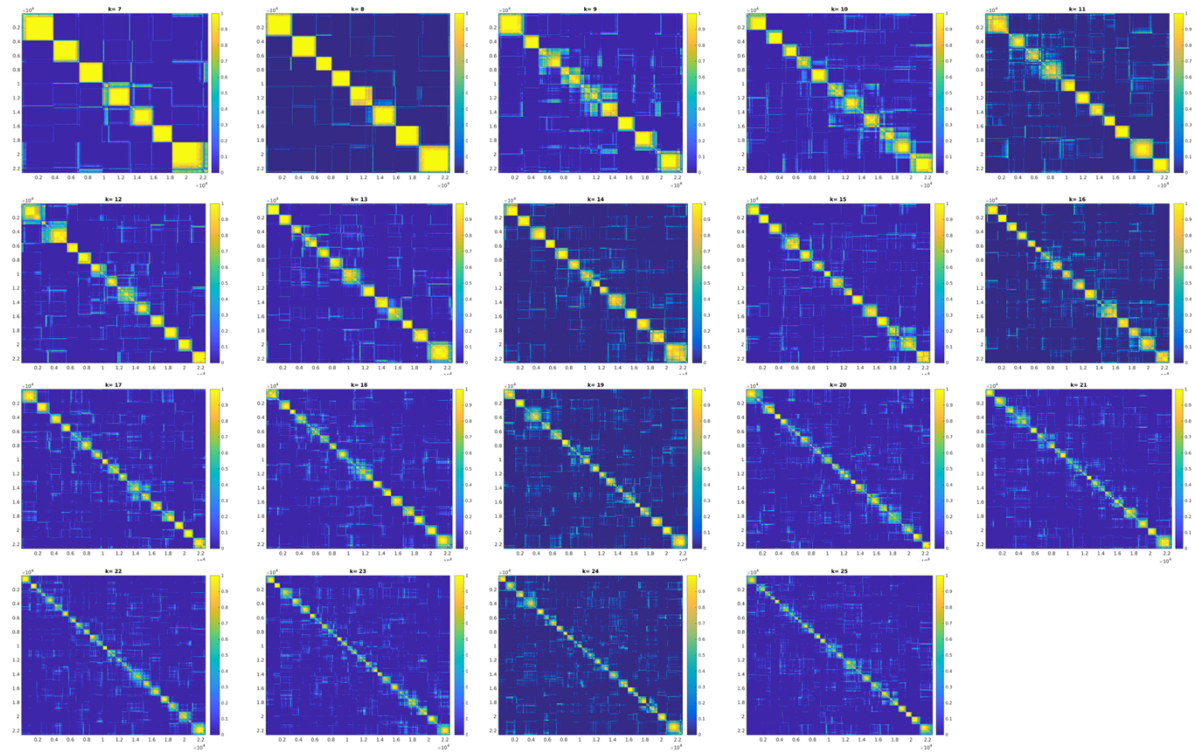

Figure S1. Consensus matrices for cluster numbers K = 7 to K = 25. High values in the matrix indicate that the two corresponding frames were clustered together during re-sampling. A value of 1 means that the frames were always clustered together, while a value of 0 means that the frames were never clustered together. We selected to use K = 13 based on visual inspection of the consensus matrices and evaluation of consensus clustering quality measures (Monti et al., 2003). In Supplementary Figure S2, we plot the distribution of average consensus values per cluster to assess the quality of the clustering for each K.

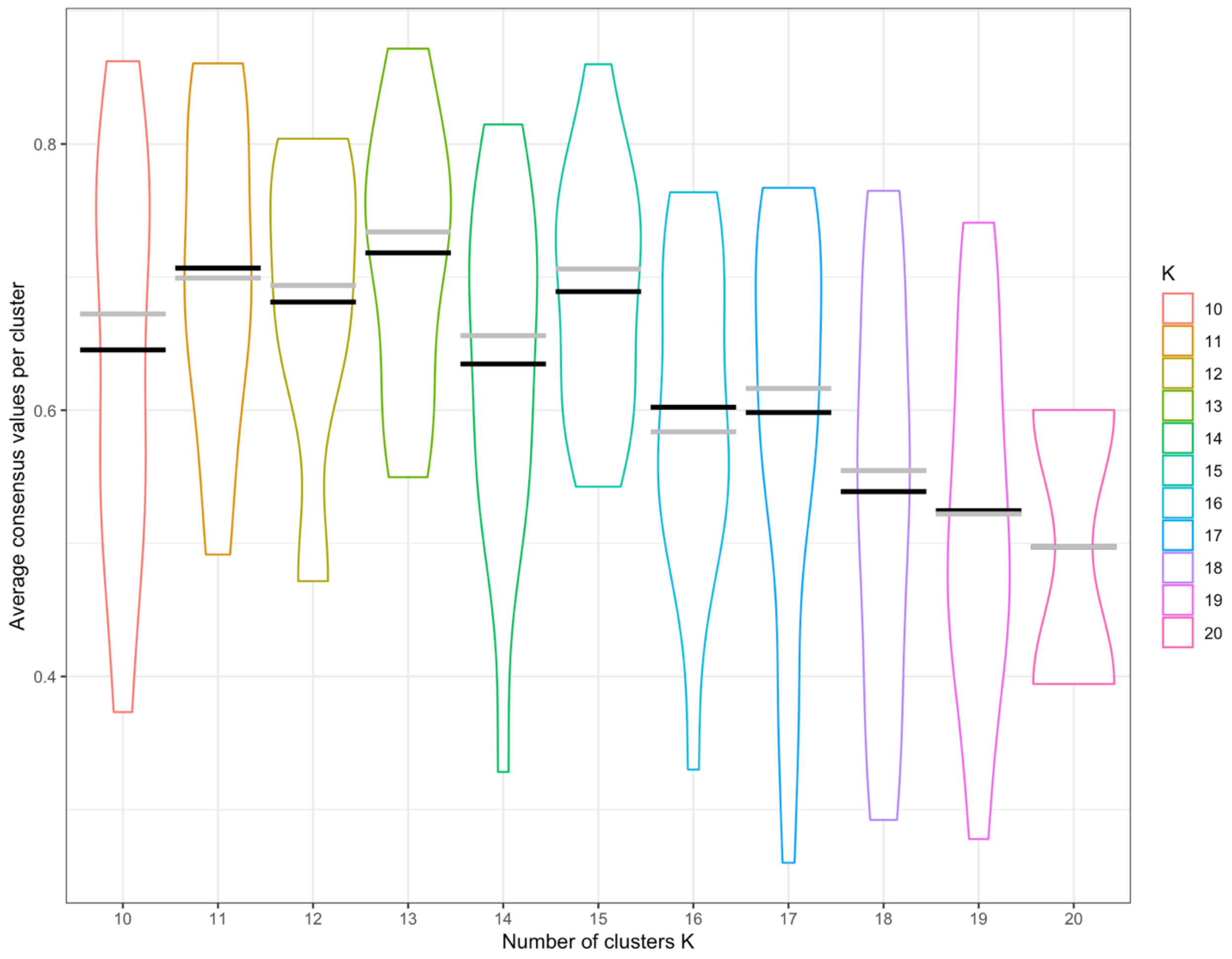

Figure S2. Consensus clustering quality measures for cluster numbers K = 10 to K = 20. The distribution of average consensus values per cluster: the mean for each cluster is represented by a black line and the median is represented by a grey line. Mean consensus is maximal (closest to 1) for K = 13.

Supplementary Tables

Table S1. iCAPs functional networks of regions from the automated anatomical labeling 2 (AAL2) atlas. Percentiles indicate the fraction of voxels of a functional network or region that have a z-score > 2.3. A network/region is listed if more than 20% of the network/region is included in the iCAP.

iCAP	Lobe	Region	Percentile	mean z-score	voxels
**iCAP 1**	Parietal	Parietal_Sup_R	64.82	2.14	457
	Parietal	Parietal_Sup_L	64.6	2.12	480
	Occipital	Occipital_Sup_L	52.4	2.11	164
	Occipital	Occipital_Sup_R	50.49	2.18	154
	Parietal	Angular_R	29.57	1.97	225
	Posterior Fossa	Cerebelum_6_R	27.52	1.8	368
	Parietal	Precuneus_L	23.9	1.92	365
	Parietal	Precuneus_R	23.21	1.84	331
	Posterior Fossa	Cerebelum_Crus1_L	19.67	1.71	302
	Posterior Fossa	Cerebelum_Crus1_R	18.27	1.72	273
	Posterior Fossa	Cerebelum_6_L	17.7	1.65	229
	Parietal	Parietal_Inf_L	16.4	1.84	208
	Occipital	Cuneus_R	14.68	1.75	74
	Occipital	Occipital_Mid_R	13.65	1.94	107
	Occipital	Occipital_Mid_L	12.91	2.05	143
	Occipital	Cuneus_L	11.24	1.78	79
	Parietal	Parietal_Inf_R	10.16	1.65	78
**iCAP 2**	Parietal	SupraMarginal_L	79.53	2.73	470
	Central	Rolandic_Oper_L	62.3	2.07	304
	Parietal	SupraMarginal_R	62.06	2.68	535
	Frontal	Rolandic_Oper_R	58.16	2.04	342
	Central	Postcentral_L	57.39	2.2	652

	Central	Precentral_R	57.39	1.96	621
	Central	Postcentral_R	49.44	2.48	613
	Temporal	Heschl_L	35.81	1.74	53
	Central	Precentral_L	35.13	1.82	372
	Frontal	Supp_Motor_Area_R	33.33	1.82	121
	Temporal	Temporal_Sup_L	30.44	2.12	309
	Frontal	Supp_Motor_Area_L	29.17	1.82	119
	Limbic	Insula_L	26.58	1.79	248
	Temporal	Heschl_R	25.76	1.68	17
	Limbic	Insula_R	24.33	1.75	236
	Limbic	Cingulate_Mid_L	21.08	1.81	219
	Limbic	Cingulate_Mid_R	20.57	1.81	224
	Parietal	Parietal_Inf_L	20.43	2.31	259
	Frontal	Frontal_Inf_Oper_L	16.27	1.75	61
	Frontal	Frontal_Inf_Oper_R	15.52	1.64	88
	Temporal	Temporal_Sup_R	10.06	1.87	104
**iCAP 3**	Posterior Fossa	Cerebelum_4_5_R	83.05	2.1	544
	Posterior Fossa	Vermis_9	81.72	1.83	76
	Posterior Fossa	Vermis_3	80.95	1.87	17
	Posterior Fossa	Cerebelum_4_5_L	75.31	2.05	540
	Posterior Fossa	Vermis_4_5	67.33	2.01	204
	Posterior Fossa	Cerebelum_9_R	62.07	1.74	36
	Posterior Fossa	Cerebelum_3_R	54.84	1.97	17
	Posterior Fossa	Vermis_8	46.58	1.63	68
	Posterior Fossa	Cerebelum_3_L	38.46	1.85	5
	Posterior Fossa	Cerebelum_9_L	36.36	1.67	20
	Posterior Fossa	Vermis_7	27.01	1.6	37
	Occipital	Fusiform_R	22.68	1.83	276

	Posterior Fossa	Vermis_6	20.97	1.65	56
	Posterior Fossa	Cerebelum_6_L	19.09	1.74	247
	Posterior Fossa	Cerebelum_6_R	15.56	1.74	208
	Occipital	Fusiform_L	14.74	1.82	175
	Occipital	Lingual_R	14.69	1.82	144
	Posterior Fossa	Vermis_1_2	12.5	1.71	1
	Occipital	Lingual_L	10.48	1.79	103
**iCAP 4**	Parietal	Parietal_Sup_L	99.06	2.51	736
	Parietal	Parietal_Sup_R	99.01	2.35	698
	Frontal	Paracentral_Lobule_R	92.68	2.02	76
	Frontal	Paracentral_Lobule_L	88.69	2.41	149
	Parietal	Parietal_Inf_R	84.11	2.03	646
	Frontal	Supp_Motor_Area_R	83.2	2.1	302
	Parietal	Parietal_Inf_L	80.84	2.02	1025
	Central	Postcentral_R	70.73	2.32	877
	Frontal	Supp_Motor_Area_L	69.85	2.11	285
	Central	Postcentral_L	51.41	2.4	584
	Central	Precentral_R	48.24	2.19	522
	Occipital	Occipital_Sup_R	43.28	1.87	132
	Parietal	Angular_R	43.23	1.93	329
	Central	Precentral_L	40.13	2.22	425
	Occipital	Occipital_Sup_L	39.3	1.89	123
	Parietal	Precuneus_L	37	2.31	565
	Frontal	Frontal_Sup_2_R	34.5	2.41	376
	Parietal	Precuneus_R	30.86	1.98	440
	Frontal	Frontal_Sup_2_L	24.28	2.3	219
	Parietal	Angular_L	21.94	1.8	129
	Limbic	Cingulate_Mid_R	20.48	1.63	223

	Parietal	SupraMarginal_L	14.72	1.73	87
	Limbic	Cingulate_Mid_L	14.53	1.59	151
	Occipital	Occipital_Mid_L	12.18	1.86	135
	Parietal	SupraMarginal_R	10.9	1.75	94
	Occipital	Cuneus_R	10.71	1.69	54
**iCAP 5**	Frontal	Frontal_Med_Orb_L	100	1.53	1
	Limbic	Cingulate_Post_L	96.28	3.15	181
	Parietal	Angular_L	92.52	2.96	544
	Limbic	Cingulate_Post_R	89.16	3.07	74
	Parietal	Angular_R	79.89	2.6	608
	Parietal	Parietal_Inf_R	70.96	2.72	545
	Parietal	Parietal_Inf_L	62.22	2.72	789
	Parietal	Precuneus_L	54.94	2.64	839
	Parietal	Precuneus_R	45.93	2.69	655
	Limbic	Cingulate_Mid_L	43.89	2.59	456
	Limbic	Cingulate_Mid_R	41.41	2.72	451
	Frontal	Frontal_Med_Orb_R	33.33	1.6	15
	Parietal	SupraMarginal_R	27.96	2.22	241
	Parietal	Parietal_Sup_L	19.92	2.22	148
	Temporal	Temporal_Mid_L	17.62	1.7	399
	Parietal	Parietal_Sup_R	15.32	2.09	108
	Parietal	SupraMarginal_L	10.49	1.91	62
	Limbic	Cingulate_Ant_L	10.08	1.6	79
**iCAP 6**	Posterior Fossa	Cerebelum_7b_R	100	2.17	46
	Posterior Fossa	Cerebelum_8_R	98.75	1.99	79
	Posterior Fossa	Cerebelum_Crus1_R	97.52	2.65	1457
	Posterior Fossa	Cerebelum_Crus1_L	95.77	2.51	1470
	Posterior Fossa	Vermis_8	95.21	1.93	139

	Posterior Fossa	Cerebelum_6_R	91.4	2.42	1222
	Posterior Fossa	Cerebelum_9_L	90.91	1.77	50
	Posterior Fossa	Cerebelum_7b_L	90.22	1.9	83
	Posterior Fossa	Cerebelum_Crus2_L	89.57	2.21	713
	Posterior Fossa	Cerebelum_Crus2_R	88.65	2.29	609
	Posterior Fossa	Vermis_7	88.32	2.21	121
	Posterior Fossa	Vermis_9	88.17	1.91	82
	Posterior Fossa	Cerebelum_6_L	82.3	2.23	1065
	Posterior Fossa	Cerebelum_8_L	81.25	1.75	52
	Posterior Fossa	Cerebelum_9_R	74.14	1.81	43
	Posterior Fossa	Vermis_6	60.67	1.91	162
	Occipital	Occipital_Inf_R	53.15	1.81	118
	Occipital	Fusiform_R	40.51	2.13	493
	Occipital	Lingual_R	36.84	1.97	361
	Occipital	Fusiform_L	33.36	2	396
	Occipital	Lingual_L	20.85	1.88	205
	Temporal	Temporal_Inf_R	13.32	1.78	151
	Occipital	Occipital_Inf_L	12.5	1.64	31
	Temporal	Temporal_Inf_L	10.61	1.75	89
**iCAP 7**	Frontal	Frontal_Inf_Tri_R	99.7	3.12	655
	Frontal	Frontal_Inf_Oper_R	99.12	2.95	562
	Central	Frontal_Inf_Orb_2_R	92.93	2.25	92
	Frontal	Frontal_Mid_2_R	91.08	2.78	1593
	Frontal	Rolandic_Oper_R	81.12	2.02	477
	Temporal	Temporal_Pole_Mid_R	78.57	1.82	22
	Parietal	SupraMarginal_R	74.83	1.86	645
	Limbic	Insula_R	73.09	2.1	709
	Temporal	Temporal_Sup_R	72.05	1.92	745

	Temporal	Temporal_Pole_Sup_R	69.19	1.97	128
	Frontal	OFCpost_R	66.67	1.55	4
	Frontal	Frontal_Sup_2_R	56.97	2.22	621
	Subcortical grey nucleus	Putamen_R	45.15	1.77	279
	Temporal	Heschl_R	43.94	1.98	29
	Parietal	Parietal_Inf_R	39.58	1.93	304
	Central	Postcentral_R	38.15	1.87	473
	Temporal	Temporal_Mid_R	36.18	1.82	683
	Central	Precentral_R	35.95	2.2	389
	Frontal	Frontal_Sup_Medial_R	20.39	1.65	73
	Temporal	Temporal_Inf_R	11.2	1.61	127
	Central	Rolandic_Oper_L	98.57	2.8	481
	Limbic	Insula_R	98.56	2.55	956
	Limbic	Insula_L	98.07	2.66	915
	Temporal	Heschl_L	97.97	2.58	145
**iCAP 8**	Frontal	Rolandic_Oper_R	97.79	2.58	575
	Temporal	Heschl_R	96.97	2.3	64
	Temporal	Temporal_Pole_Sup_R	92.97	2.35	172
	Temporal	Temporal_Pole_Mid_R	92.86	2.03	26
	Temporal	Temporal_Pole_Sup_L	90.07	2.28	127
	Subcortical grey nucleus	Putamen_R	86.89	2.17	537
	Subcortical grey nucleus	Putamen_L	83.92	2.21	407
	Frontal	OFCpost_R	83.33	1.83	5
	Subcortical grey nucleus	Amygdala_R	82.41	1.95	89
	Temporal	Temporal_Sup_L	80.99	2.23	822
	Frontal	Frontal_Inf_Orb_2_L	80.67	1.99	121
	Central	Frontal_Inf_Orb_2_R	77.78	1.94	77
	Subcortical grey nucleus	Amygdala_L	62.6	1.86	77

	Frontal	OFCpost_L	62.5	1.69	5
	Limbic	Hippocampus_R	53.23	1.77	107
	Subcortical grey nucleus	Pallidum_R	51.85	1.87	28
	Temporal	Temporal_Sup_R	50.39	2.02	521
	Frontal	Frontal_Inf_Tri_R	46.58	1.99	306
	Frontal	Frontal_Inf_Oper_L	44.53	2.05	167
	Frontal	Frontal_Inf_Tri_L	38.62	2.09	297
	Frontal	Frontal_Inf_Oper_R	34.04	2.26	193
	Frontal	Olfactory_R	28.67	1.91	43
	Limbic	Hippocampus_L	28.02	1.7	72
	Limbic	ParaHippocampal_R	25.97	1.78	120
	Parietal	SupraMarginal_L	23.01	2.16	136
	Subcortical grey nucleus	Pallidum_L	21.74	1.78	10
	Parietal	SupraMarginal_R	17.52	2.03	151
	Frontal	Olfactory_L	16.33	1.88	16
	Subcortical grey nucleus	Caudate_R	13.39	1.61	32
	Central	Postcentral_L	10.56	2.22	120
**iCAP 9**	Frontal	Frontal_Inf_Tri_L	99.22	2.76	763
	Frontal	Frontal_Inf_Oper_L	98.93	2.67	371
	Frontal	Frontal_Mid_2_L	95.41	2.33	1434
	Frontal	Frontal_Inf_Orb_2_L	95.33	2.16	143
	Central	Rolandic_Oper_L	91.8	1.92	448
	Parietal	SupraMarginal_L	88.16	1.99	521
	Temporal	Temporal_Sup_L	87.68	2.13	890
	Temporal	Temporal_Pole_Sup_L	79.43	2.16	112
	Limbic	Insula_L	78.03	1.91	728
	Temporal	Heschl_L	75.68	1.84	112
	Frontal	OFCpost_L	75	1.69	6

	Temporal	Temporal_Mid_L	69.52	2.15	1574
	Central	Precentral_L	66.1	2.51	700
	Frontal	Frontal_Sup_2_L	64.19	1.89	579
	Parietal	Parietal_Inf_L	53.71	1.84	681
	Central	Postcentral_L	52.64	1.93	598
	Parietal	Angular_L	47.45	1.83	279
	Subcortical grey nucleus	Putamen_L	47.01	1.71	228
	Frontal	Frontal_Sup_Medial_L	22.36	1.75	108
	Temporal	Temporal_Inf_L	21.1	1.68	177
**iCAP 10**	Occipital	Occipital_Mid_R	88.39	3.1	693
	Occipital	Occipital_Mid_L	82.58	3.1	915
	Occipital	Occipital_Inf_L	71.77	2.34	178
	Temporal	Temporal_Mid_R	55.56	2.79	1049
	Occipital	Occipital_Inf_R	49.55	2.02	110
	Occipital	Occipital_Sup_L	43.77	2.35	137
	Temporal	Temporal_Mid_L	41.03	2.31	929
	Occipital	Occipital_Sup_R	31.15	2.26	95
	Occipital	Cuneus_L	27.45	1.79	193
	Temporal	Temporal_Sup_R	27.27	2.05	282
	Parietal	Angular_R	26.68	1.97	203
	Parietal	Angular_L	21.77	1.89	128
	Temporal	Temporal_Inf_R	21.16	2.09	240
	Occipital	Cuneus_R	18.45	1.74	93
	Occipital	Calcarine_L	11.05	1.69	121
	Temporal	Temporal_Sup_L	11.03	1.85	112
	Temporal	Temporal_Inf_L	10.25	1.93	86
	Posterior Fossa	Cerebelum_Crus2_R	10.19	1.64	70
	Occipital	Calcarine_R	10.06	1.57	63

**iCAP 11**	Occipital	Cuneus_L	99.86	2.98	702
	Occipital	Cuneus_R	94.84	2.61	478
	Occipital	Calcarine_R	94.09	3.62	589
	Occipital	Occipital_Sup_L	84.66	2.06	265
	Occipital	Calcarine_L	81.28	3.46	890
	Occipital	Lingual_L	69.68	2.48	685
	Occipital	Lingual_R	55.2	2.33	541
	Occipital	Occipital_Sup_R	55.08	1.82	168
	Occipital	Occipital_Mid_R	43.49	1.76	341
	Parietal	Precuneus_R	43.27	2.79	617
	Occipital	Occipital_Mid_L	42.96	1.88	476
	Parietal	Precuneus_L	31.83	2.55	486
	Posterior Fossa	Cerebelum_4_5_L	11.44	1.92	82
**iCAP 12**	Frontal	Frontal_Med_Orb_L	100	4.19	1
	Frontal	Frontal_Med_Orb_R	95.56	3.19	43
	Limbic	Cingulate_Ant_R	94.95	3.05	470
	Frontal	Frontal_Sup_Medial_R	88.27	3.05	316
	Limbic	Cingulate_Ant_L	86.73	3.07	680
	Frontal	Frontal_Sup_Medial_L	79.5	2.6	384
	Frontal	Frontal_Inf_Orb_2_L	75.33	1.84	113
	Frontal	Frontal_Sup_2_L	65.96	3.04	595
	Frontal	Frontal_Inf_Tri_L	65.15	2.21	501
	Frontal	Frontal_Mid_2_L	63.87	2.68	960
	Frontal	Frontal_Mid_2_R	61.58	2.64	1077
	Frontal	Frontal_Sup_2_R	54.5	3.34	594
	Frontal	Frontal_Inf_Tri_R	49.01	2.1	322
	Central	Frontal_Inf_Orb_2_R	40.4	2.22	40
**iCAP 13**	Posterior Fossa	Cerebelum_Crus2_R	87.19	3.47	599

Posterior Fossa	Cerebelum_Crus2_L	86.06	3.58	685
Posterior Fossa	Vermis_8	84.93	2.74	124
Occipital	Occipital_Inf_R	79.28	2.27	176
Posterior Fossa	Cerebelum_8_L	67.19	2.09	43
Posterior Fossa	Cerebelum_8_R	66.25	2.03	53
Occipital	Occipital_Inf_L	63.31	2.25	157
Posterior Fossa	Cerebelum_7b_R	63.04	2.22	29
Posterior Fossa	Vermis_7	53.28	2.02	73
Posterior Fossa	Cerebelum_7b_L	53.26	2.77	49
Posterior Fossa	Cerebelum_Crus1_R	52.28	2.94	781
Posterior Fossa	Cerebelum_Crus1_L	51.99	2.83	798
Posterior Fossa	Vermis_9	38.71	2.17	36
Temporal	Temporal_Inf_R	27.95	1.85	317
Posterior Fossa	Cerebelum_9_L	27.27	1.86	15
Posterior Fossa	Cerebelum_9_R	24.14	2.09	14
Occipital	Lingual_R	20.2	2.57	198
Posterior Fossa	Vermis_6	18.73	1.99	50
Occipital	Calcarine_L	17.72	2.14	194
Temporal	Temporal_Inf_L	17.16	1.8	144
Occipital	Occipital_Mid_L	13.9	1.89	154
Occipital	Fusiform_L	12.97	2.06	154
Occipital	Lingual_L	12.11	2.71	119

Table S1.a. Dice coefficient of similarity between the 13 icaps retrieved and the Yeo's 17 cortical resting-state network.[45]

	N1: VisCent	N2: VisPeri	N3: SomMotA	N4: SomMotB	N5: DorsAttnA	N6: DorsAttnB	N7: SalVentAttnA	N8: SalVentAttnB	N9: Limbic B	N10: Limbic A	N11: ContA	N12: ContB	N13: ContC	N14: Default A	N15: Default B	N16: Default C	N17: TempPar	N1: VisCent
icap 1 - precuneus/posterior default mode network (DMN)	0.003	0.007	0.000	0.000	0.152	0.013	0.000	0.000	0.000	0.000	0.050	0.042	0.003	0.000	0.004	0.002	0.000	0.003
icap 2 - sensori-motor/auditory (AUD)	0.000	0.000	0.066	0.161	0.000	0.100	0.157	0.018	0.000	0.000	0.000	0.005	0.001	0.004	0.000	0.000	0.000	0.000
icap 3 - anterior cerebellum/vermis	0.011	0.032	0.000	0.000	0.003	0.000	0.000	0.000	0.000	0.000	0.000	0.000	0.000	0.000	0.004	0.000	0.000	0.011
icap 4 - fronto-parietal > Dorsal Attention	0.000	0.001	0.203	0.000	0.149	0.220	0.016	0.006	0.000	0.000	0.023	0.105	0.020	0.001	0.001	0.007	0.002	0.000
icap 5- posterior default mode network (pDMN) > executive control	0.000	0.000	0.001	0.000	0.021	0.004	0.005	0.008	0.000	0.000	0.204	0.096	0.085	0.000	0.000	0.193	0.025	0.000
icap 6- posterior cerebellum	0.108	0.001	0.000	0.000	0.006	0.000	0.000	0.000	0.000	0.000	0.000	0.000	0.000	0.000	0.000	0.000	0.000	0.108
icap 7- fronto-temporal right	0.000	0.000	0.003	0.047	0.002	0.036	0.086	0.177	0.000	0.000	0.002	0.120	0.125	0.051	0.000	0.008	0.034	0.000
icap 8 - anterior insula/amygdala	0.000	0.000	0.006	0.290	0.000	0.000	0.205	0.093	0.003	0.000	0.000	0.001	0.001	0.043	0.000	0.001	0.035	0.000
icap 9 - fronto-temporal left	0.000	0.000	0.004	0.090	0.000	0.039	0.057	0.116	0.002	0.000	0.000	0.123	0.059	0.130	0.000	0.014	0.147	0.000
icap 10 - secondary visual	0.153	0.012	0.000	0.000	0.188	0.034	0.005	0.001	0.000	0.000	0.000	0.007	0.000	0.098	0.069	0.032	0.009	0.153
icap 11 - primary visual > Visual Peripheric	0.037	0.375	0.000	0.000	0.009	0.000	0.000	0.000	0.000	0.000	0.154	0.000	0.000	0.000	0.090	0.045	0.000	0.037
icap 12 - frontal	0.000	0.000	0.000	0.000	0.000	0.000	0.000	0.255	0.000	0.002	0.000	0.079	0.031	0.001	0.000	0.125	0.056	0.000
icap 13 - cerebellum/visual	0.116	0.000	0.000	0.000	0.019	0.000	0.000	0.000	0.000	0.000	0.000	0.006	0.000	0.000	0.000	0.001	0.000	0.116

Note: The Network parcellation of the Yeo's 17 networks include the following regions: N1: VisCent-Visual A; N2: VisPeri-Visual B; N3: SomMotA-Somatomotor A; N4: SomMotB-Somatomotor B; N5: DorsAttnA-Dorsal Attention A; N6: DorsAttnB-Dorsal Attention B; N7: SalVentAttnA-Salience/Ventral Attention A; N8: SalVentAttnB-

Salience/Ventral Attention B; N9: LimbicB-Limbic B; N10: LimbicA-Limbic A; N11: ContA-Control A; N12: ContB-Control B; N13: ContC-Control C; N14: DefaultA-Default A; N15: DefaultB-Default B; N16: DefaultC-Default C; and N17: TempPar-Temporal Parietal.

Table S2. Group comparison for the VPT and full-term control groups for the occurrence of the identified iCAPs networks using a general linear model including socio-economic status (based on the Largo score) and sex as covariate.

	VPT group		Full-term group		General linear models (including socio-economic status and sex as covariate)		
**iCAPs – Occurrence**	**Mean**	**SD**	**Mean**	**SD**	**F**	**p-value**	**q-value (FDR)**
iCAP 1	34.679	5.863	34.333	3.774	F (1,36) = 0.029	0.865	0.937
iCAP 2	28.179	6.301	28.583	4.814	F (1,36) =3.236	0.080	0.523
iCAP 3	24.179	6.037	24.583	7.879	F (1,36) =0.571	0.455	0.845
iCAP 4	17.286	3.670	17.333	4.559	F (1,36) =0.003	0.985	0.985
iCAP 5	20.357	6.442	24.083	6.288	F (1,36) =6.333	0.016	0.214
iCAP 6	23.321	6.481	24.750	4.159	F (1,36) =1.353	0.252	0.671
iCAP 7	17.393	4.856	17.000	5.908	F (1,36) =0.094	0.761	0.899
iCAP 8	14.893	5.370	13.333	5.483	F (1,36) =0.175	0.678	0.899
iCAP 9	20.214	5.776	21.667	5.416	F (1,36) =0.812	0.373	0.809
iCAP 10	17.000	5.185	17.917	5.485	F (1,36) =1.32	0.258	0.671
iCAP 11	8.750	4.070	8.167	5.167	F (1,36) =0.267	0.609	0.899
iCAP 12	9.250	4.291	7.500	5.018	F (1,36) =2.211	0.146	0.631
iCAP 13	4.500	2.975	3.917	1.505	F (1,36) =0.097	0.757	0.899

Table S3. Group comparison for the VPT and full-term control groups for the total duration of the identified iCAPs networks using a general linear model including socio-economic status (based on the Largo score) and sex as covariate.

iCAPs – Total duration	VPT group		Full-term group		General linear models (including socio-economic status and sex as covariate)		
	Mean	SD	Mean	SD	F	p-value	q-value (FDR)
iCAP 1	44.477	8.636	49.705	8.020	F (1,36) = 3.491	0.070	0.316
iCAP 2	32.072	9.216	33.036	7.019	F (1,36) =0.545	0.465	0.609
iCAP 3	21.889	5.857	20.643	5.536	F (1,36) =0.046	0.831	0.831
iCAP 4	19.855	4.991	17.964	6.581	F (1,36) =1.558	0.220	0.609
iCAP 5	24.793	10.264	29.196	8.310	F (1,36) =3.411	0.073	0.316
iCAP 6	23.950	7.687	26.015	5.529	F (1,36) =1.218	0.277	0.609
iCAP 7	23.661	6.349	20.995	6.528	F (1,36) =0.811	0.374	0.609
iCAP 8	15.501	7.707	12.746	5.295	F (1,36) =0.317	0.577	0.682
iCAP 9	23.614	7.244	27.313	7.637	F (1,36) =1.091	0.303	0.609
iCAP 10	17.618	6.518	17.781	5.031	F (1,36) =0.112	0.740	0.801
iCAP 11	10.408	6.744	9.193	6.657	F (1,36) =0.538	0.468	0.609
iCAP 12	8.360	4.815	5.876	3.488	F (1,36) =4.766	0.036	0.316
iCAP 13	4.248	3.084	3.334	2.205	F (1,36) =0.633	0.431	0.609

Table S4. Group comparison for the VPT and full-term control groups for the coupling of the identified iCAPs networks using a general linear model including socio-economic status (based on the Largo score) and sex as covariate.

Coupling	VPT group		Full-term group		General linear models (including socio-economic status and sex as covariate)		
	Mean	SD	Mean	SD	F	p-value	q-value (FDR)
iCAP1 & iCAP2	0.116	0.068	0.121	0.054	F (1,36) = 0.023	0.881	0.975
iCAP1 & iCAP3	0.09	0.046	0.101	0.042	F (1,36) = 1.068	0.308	0.793
iCAP1 & iCAP4	0.03	0.029	0.032	0.025	F (1,36) = 0.597	0.445	0.793
iCAP1 & iCAP5	0.064	0.025	0.067	0.039	F (1,36) = 0.005	0.946	0.975
iCAP1 & iCAP6	0.044	0.027	0.022	0.015	F (1,36) = 4.669	0.037	0.372
iCAP1 & iCAP7	0.163	0.076	0.147	0.053	F (1,36) = 0.005	0.946	0.975
iCAP1 & iCAP8	0.075	0.046	0.065	0.04	F (1,36) = 0.099	0.754	0.925
iCAP1 & iCAP9	0.156	0.048	0.148	0.068	F (1,36) = 0.469	0.498	0.793
iCAP1 & iCAP10	0.071	0.042	0.039	0.025	F (1,36) = 10.757	0.002	0.090
iCAP1 & iCAP11	0.05	0.048	0.05	0.042	F (1,36) = 0.07	0.792	0.951
iCAP1 & iCAP12	0.064	0.05	0.049	0.026	F (1,36) = 1.737	0.196	0.793
iCAP1 & iCAP13	0.042	0.044	0.05	0.036	F (1,36) = 0.054	0.818	0.967
iCAP2 & iCAP3	0.101	0.059	0.103	0.054	F (1,36) = 0.107	0.745	0.925
iCAP2 & iCAP4	0.116	0.068	0.121	0.054	F (1,36) = 0.964	0.333	0.793
iCAP2 & iCAP5	0.09	0.046	0.101	0.042	F (1,36) = 0.039	0.845	0.969
iCAP2 & iCAP6	0.03	0.029	0.032	0.025	F (1,36) = 0.381	0.541	0.796
iCAP2 & iCAP7	0.064	0.025	0.067	0.039	F (1,36) = 0.357	0.554	0.797
iCAP2 & iCAP8	0.044	0.027	0.022	0.015	F (1,36) = 0.933	0.341	0.793
iCAP2 & iCAP9	0.163	0.076	0.147	0.053	F (1,36) = 0.343	0.562	0.797
iCAP2 & iCAP10	0.075	0.046	0.065	0.04	F (1,36) = 0.746	0.394	0.793
iCAP2 & iCAP11	0.156	0.048	0.148	0.068	F (1,36) = 1.555	0.220	0.793
iCAP2 & iCAP12	0.071	0.042	0.039	0.025	F (1,36) = 5.352	0.027	0.345
iCAP2 & iCAP13	0.05	0.048	0.05	0.042	F (1,36) = 9.754	0.004	0.092
iCAP3 & iCAP4	0.064	0.05	0.049	0.026	F (1,36) = 2.574	0.117	0.704

iCAP3 & iCAP5	0.042	0.044	0.05	0.036	F (1,36) = 1.892	0.177	0.793
iCAP3 & iCAP6	0.101	0.059	0.103	0.054	F (1,36) = 0.952	0.336	0.793
iCAP3 & iCAP7	0.116	0.068	0.121	0.054	F (1,36) = 0	0.999	0.999
iCAP3 & iCAP8	0.09	0.046	0.101	0.042	F (1,36) = 0.174	0.679	0.898
iCAP3 & iCAP9	0.03	0.029	0.032	0.025	F (1,36) = 0.578	0.452	0.793
iCAP3 & iCAP10	0.064	0.025	0.067	0.039	F (1,36) = 0.01	0.920	0.975
iCAP3 & iCAP11	0.044	0.027	0.022	0.015	F (1,36) = 0.006	0.940	0.975
iCAP3 & iCAP12	0.163	0.076	0.147	0.053	F (1,36) = 8.109	0.007	0.141
iCAP3 & iCAP13	0.075	0.046	0.065	0.04	F (1,36) = 0.768	0.387	0.793
iCAP4 & iCAP5	0.156	0.048	0.148	0.068	F (1,36) = 0.263	0.611	0.851
iCAP4 & iCAP6	0.071	0.042	0.039	0.025	F (1,36) = 0.437	0.513	0.793
iCAP4 & iCAP7	0.05	0.048	0.05	0.042	F (1,36) = 4.62	0.038	0.372
iCAP4 & iCAP8	0.064	0.05	0.049	0.026	F (1,36) = 1.624	0.211	0.793
iCAP4 & iCAP9	0.042	0.044	0.05	0.036	F (1,36) = 0.688	0.412	0.793
iCAP4 & iCAP10	0.101	0.059	0.103	0.054	F (1,36) = 0.701	0.408	0.793
iCAP4 & iCAP11	0.116	0.068	0.121	0.054	F (1,36) = 0.216	0.645	0.881
iCAP4 & iCAP12	0.09	0.046	0.101	0.042	F (1,36) = 1.189	0.283	0.793
iCAP4 & iCAP13	0.03	0.029	0.032	0.025	F (1,36) = 1.484	0.231	0.793
iCAP5 & iCAP6	0.064	0.025	0.067	0.039	F (1,36) = 2.121	0.154	0.793
iCAP5 & iCAP7	0.044	0.027	0.022	0.015	F (1,36) = 2.912	0.097	0.627
iCAP5 & iCAP8	0.163	0.076	0.147	0.053	F (1,36) = 0.447	0.508	0.793
iCAP5 & iCAP9	0.075	0.046	0.065	0.04	F (1,36) = 0.405	0.528	0.793
iCAP5 & iCAP10	0.156	0.048	0.148	0.068	F (1,36) = 1.683	0.203	0.793
iCAP5 & iCAP11	0.071	0.042	0.039	0.025	F (1,36) = 0.621	0.436	0.793
iCAP5 & iCAP12	0.05	0.048	0.05	0.042	F (1,36) = 0.001	0.975	0.988
iCAP5 & iCAP13	0.064	0.05	0.049	0.026	F (1,36) = 1.128	0.295	0.793
iCAP6 & iCAP7	0.042	0.044	0.05	0.036	F (1,36) = 1.635	0.209	0.793
iCAP6 & iCAP8	0.101	0.059	0.103	0.054	F (1,36) = 0.118	0.733	0.925
iCAP6 & iCAP9	0.116	0.068	0.121	0.054	F (1,36) = 0.203	0.655	0.881

iCAP6 & iCAP10	0.09	0.046	0.101	0.042	F (1,36) = 14.641	0.000	0.039
iCAP6 & iCAP11	0.03	0.029	0.032	0.025	F (1,36) = 5.724	0.022	0.344
iCAP6 & iCAP12	0.064	0.025	0.067	0.039	F (1,36) = 2.278	0.140	0.780
iCAP6 & iCAP13	0.044	0.027	0.022	0.015	F (1,36) = 0.774	0.385	0.793
iCAP7 & iCAP8	0.163	0.076	0.147	0.053	F (1,36) = 0.426	0.518	0.793
iCAP7 & iCAP9	0.075	0.046	0.065	0.04	F (1,36) = 0.012	0.915	0.975
iCAP7 & iCAP10	0.156	0.048	0.148	0.068	F (1,36) = 0.63	0.432	0.793
iCAP7 & iCAP11	0.071	0.042	0.039	0.025	F (1,36) = 1.296	0.262	0.793
iCAP7 & iCAP12	0.05	0.048	0.05	0.042	F (1,36) = 0.565	0.457	0.793
iCAP7 & iCAP13	0.064	0.05	0.049	0.026	F (1,36) = 0.096	0.759	0.925
iCAP8 & iCAP9	0.042	0.044	0.05	0.036	F (1,36) = 0.04	0.843	0.969
iCAP8 & iCAP10	0.101	0.059	0.103	0.054	F (1,36) = 4.406	0.043	0.372
iCAP8 & iCAP11	0.116	0.068	0.121	0.054	F (1,36) = 0.012	0.914	0.975
iCAP8 & iCAP12	0.09	0.046	0.101	0.042	F (1,36) = 0.681	0.415	0.793
iCAP8 & iCAP13	0.03	0.029	0.032	0.025	F (1,36) = 0.797	0.378	0.793
iCAP9 & iCAP10	0.064	0.025	0.067	0.039	F (1,36) = 0.69	0.412	0.793
iCAP9 & iCAP11	0.044	0.027	0.022	0.015	F (1,36) = 1.241	0.273	0.793
iCAP9 & iCAP12	0.163	0.076	0.147	0.053	F (1,36) = 3.898	0.056	0.437
iCAP9 & iCAP13	0.075	0.046	0.065	0.04	F (1,36) = 0.004	0.950	0.975
iCAP10 & iCAP11	0.156	0.048	0.148	0.068	F (1,36) = 0.51	0.480	0.793
iCAP10 & iCAP12	0.071	0.042	0.039	0.025	F (1,36) = 0.484	0.491	0.793
iCAP10 & iCAP13	0.05	0.048	0.05	0.042	F (1,36) = 3.041	0.090	0.627
iCAP11 & iCAP12	0.064	0.05	0.049	0.026	F (1,36) = 0.484	0.491	0.793
iCAP11 & iCAP13	0.042	0.044	0.05	0.036	F (1,36) = 1.436	0.239	0.793
iCAP12 & iCAP13	0.101	0.059	0.103	0.054	F (1,36) = 0.135	0.716	0.925

Table S5. Group comparison for the VPT and full-term control groups for the anti-coupling of the identified iCAPs networks using a general linear model including socio-economic status (based on the Largo score) and sex as covariate.

Anti-coupling	VPT group		Full-term group		General linear models (including socio-economic status and sex as covariate)		
	Mean	SD	Mean	SD	F	p-value	q-value (FDR)
iCAP1 & iCAP2	0.161	0.069	0.155	0.068	F (1,36) = 0.313	0.579	0.869
iCAP1 & iCAP3	0.118	0.041	0.124	0.065	F (1,36) = 0.00005	0.994	0.995
iCAP1 & iCAP4	0.236	0.071	0.215	0.117	F (1,36) = 0.668	0.419	0.743
iCAP1 & iCAP5	0.176	0.086	0.200	0.063	F (1,36) = 1.374	0.249	0.740
iCAP1 & iCAP6	0.239	0.081	0.305	0.090	F (1,36) = 4.832	0.034	0.526
iCAP1 & iCAP7	0.078	0.066	0.063	0.037	F (1,36) = 2.521	0.121	0.647
iCAP1 & iCAP8	0.084	0.048	0.083	0.047	F (1,36) = 0.008	0.928	0.995
iCAP1 & iCAP9	0.075	0.044	0.087	0.041	F (1,36) = 0.003	0.959	0.995
iCAP1 & iCAP10	0.117	0.071	0.162	0.059	F (1,36) = 4.789	0.035	0.526
iCAP1 & iCAP11	0.081	0.067	0.062	0.046	F (1,36) = 2.096	0.156	0.647
iCAP1 & iCAP12	0.040	0.038	0.027	0.027	F (1,36) = 1.921	0.174	0.647
iCAP1 & iCAP13	0.020	0.023	0.010	0.015	F (1,36) = 1.984	0.168	0.647
iCAP2 & iCAP3	0.111	0.052	0.121	0.100	F (1,36) = 0.0001	0.990	0.995
iCAP2 & iCAP4	0.161	0.069	0.155	0.068	F (1,36) = 4.389	0.043	0.526
iCAP2 & iCAP5	0.118	0.041	0.124	0.065	F (1,36) = 0.131	0.719	0.985
iCAP2 & iCAP6	0.236	0.071	0.215	0.117	F (1,36) = 1.089	0.304	0.740
iCAP2 & iCAP7	0.176	0.086	0.200	0.063	F (1,36) = 0.014	0.905	0.995
iCAP2 & iCAP8	0.239	0.081	0.305	0.090	F (1,36) = 4.182	0.048	0.526
iCAP2 & iCAP9	0.078	0.066	0.063	0.037	F (1,36) = 0.188	0.667	0.941
iCAP2 & iCAP10	0.084	0.048	0.083	0.047	F (1,36) = 2.527	0.121	0.647
iCAP2 & iCAP11	0.075	0.044	0.087	0.041	F (1,36) = 0.001	0.976	0.995
iCAP2 & iCAP12	0.117	0.071	0.162	0.059	F (1,36) = 0.705	0.407	0.743
iCAP2 & iCAP13	0.081	0.067	0.062	0.046	F (1,36) = 1.156	0.289	0.740

iCAP3 & iCAP4	0.040	0.038	0.027	0.027	F (1,36) = 0.034	0.854	0.995
iCAP3 & iCAP5	0.020	0.023	0.010	0.015	F (1,36) = 0.693	0.411	0.743
iCAP3 & iCAP6	0.111	0.052	0.121	0.100	F (1,36) = 0.775	0.384	0.743
iCAP3 & iCAP7	0.161	0.069	0.155	0.068	F (1,36) = 0.705	0.407	0.743
iCAP3 & iCAP8	0.118	0.041	0.124	0.065	F (1,36) = 0.034	0.855	0.995
iCAP3 & iCAP9	0.236	0.071	0.215	0.117	F (1,36) = 0.179	0.675	0.941
iCAP3 & iCAP10	0.176	0.086	0.200	0.063	F (1,36) = 0.001	0.970	0.995
iCAP3 & iCAP11	0.239	0.081	0.305	0.090	F (1,36) = 1.097	0.302	0.740
iCAP3 & iCAP12	0.078	0.066	0.063	0.037	F (1,36) = 0.075	0.786	0.995
iCAP3 & iCAP13	0.084	0.048	0.083	0.047	F (1,36) = 0.936	0.340	0.743
iCAP4 & iCAP5	0.075	0.044	0.087	0.041	F (1,36) = 0.001	0.970	0.995
iCAP4 & iCAP6	0.117	0.071	0.162	0.059	F (1,36) = 2.713	0.108	0.647
iCAP4 & iCAP7	0.081	0.067	0.062	0.046	F (1,36) = 0.043	0.837	0.995
iCAP4 & iCAP8	0.040	0.038	0.027	0.027	F (1,36) = 0.762	0.388	0.743
iCAP4 & iCAP9	0.020	0.023	0.010	0.015	F (1,36) = 0.53	0.471	0.799
iCAP4 & iCAP10	0.111	0.052	0.121	0.100	F (1,36) = 1.494	0.230	0.740
iCAP4 & iCAP11	0.161	0.069	0.155	0.068	F (1,36) = 1.222	0.276	0.740
iCAP4 & iCAP12	0.118	0.041	0.124	0.065	F (1,36) = 0.984	0.328	0.743
iCAP4 & iCAP13	0.236	0.071	0.215	0.117	F (1,36) = 1.149	0.291	0.740
iCAP5 & iCAP6	0.176	0.086	0.200	0.063	F (1,36) = 2.107	0.155	0.647
iCAP5 & iCAP7	0.239	0.081	0.305	0.090	F (1,36) = 2.448	0.126	0.647
iCAP5 & iCAP8	0.078	0.066	0.063	0.037	F (1,36) = 1.382	0.247	0.740
iCAP5 & iCAP9	0.084	0.048	0.083	0.047	F (1,36) = 3.554	0.067	0.526
iCAP5 & iCAP10	0.075	0.044	0.087	0.041	F (1,36) = 0.321	0.574	0.869
iCAP5 & iCAP11	0.117	0.071	0.162	0.059	F (1,36) = 0.083	0.775	0.995
iCAP5 & iCAP12	0.081	0.067	0.062	0.046	F (1,36) = 0.763	0.388	0.743
iCAP5 & iCAP13	0.040	0.038	0.027	0.027	F (1,36) = 8.242	0.007	0.292
iCAP6 & iCAP7	0.020	0.023	0.010	0.015	F (1,36) = 0.446	0.508	0.826
iCAP6 & iCAP8	0.111	0.052	0.121	0.100	F (1,36) = 0.97	0.331	0.743

iCAP6 & iCAP9	0.161	0.069	0.155	0.068	F (1,36) = 3.342	0.076	0.538
iCAP6 & iCAP10	0.118	0.041	0.124	0.065	F (1,36) = 8.034	0.007	0.292
iCAP6 & iCAP11	0.236	0.071	0.215	0.117	F (1,36) = 0.099	0.755	0.995
iCAP6 & iCAP12	0.176	0.086	0.200	0.063	F (1,36) = 0.783	0.382	0.743
iCAP6 & iCAP13	0.239	0.081	0.305	0.090	F (1,36) = 0.294	0.591	0.870
iCAP7 & iCAP8	0.078	0.066	0.063	0.037	F (1,36) = 0.487	0.490	0.813
iCAP7 & iCAP9	0.084	0.048	0.083	0.047	F (1,36) = 1.606	0.213	0.740
iCAP7 & iCAP10	0.075	0.044	0.087	0.041	F (1,36) = 0.00004	0.995	0.995
iCAP7 & iCAP11	0.117	0.071	0.162	0.059	F (1,36) = 2.071	0.159	0.647
iCAP7 & iCAP12	0.081	0.067	0.062	0.046	F (1,36) = 3.925	0.055	0.526
iCAP7 & iCAP13	0.040	0.038	0.027	0.027	F (1,36) = 0.036	0.851	0.995
iCAP8 & iCAP9	0.020	0.023	0.010	0.015	F (1,36) = 0.599	0.444	0.770
iCAP8 & iCAP10	0.111	0.052	0.121	0.100	F (1,36) = 0.085	0.772	0.995
iCAP8 & iCAP11	0.161	0.069	0.155	0.068	F (1,36) = 1.304	0.261	0.740
iCAP8 & iCAP12	0.118	0.041	0.124	0.065	F (1,36) = 1.982	0.168	0.647
iCAP8 & iCAP13	0.236	0.071	0.215	0.117	F (1,36) = 0.178	0.675	0.941
iCAP9 & iCAP10	0.176	0.086	0.200	0.063	F (1,36) = 1.229	0.275	0.740
iCAP9 & iCAP11	0.239	0.081	0.305	0.090	F (1,36) = 0.015	0.902	0.995
iCAP9 & iCAP12	0.078	0.066	0.063	0.037	F (1,36) = 3.648	0.064	0.526
iCAP9 & iCAP13	0.084	0.048	0.083	0.047	F (1,36) = 0.003	0.958	0.995
iCAP10 & iCAP11	0.075	0.044	0.087	0.041	F (1,36) = 0.009	0.924	0.995
iCAP10 & iCAP12	0.117	0.071	0.162	0.059	F (1,36) = 6.647	0.014	0.368
iCAP10 & iCAP13	0.081	0.067	0.062	0.046	F (1,36) = 0.361	0.552	0.861
iCAP11 & iCAP12	0.040	0.038	0.027	0.027	F (1,36) = 0.403	0.530	0.843
iCAP11 & iCAP13	0.020	0.023	0.010	0.015	F (1,36) = 0.755	0.391	0.743
iCAP12 & iCAP13	0.111	0.052	0.121	0.100	F (1,36) = 0.06	0.809	0.995

Table S6. Group comparison for the VPT and full-term control groups for the Amplitude-Laterality Index (A-LI) of the identified iCAPs networks using a general linear model including hand preference, socio-economic status (based on the Largo score) and sex as covariates.

**Amplitude-LI**	**VPT group**		**Full-term group**		**General linear model including hand preference, socio-economic status and sex as covariates**		
	**Mean**	**SD**	**Mean**	**SD**	**F**	**p-value**	**q-value (FDR)**
iCAP 1	0.027	0.034	0.030	0.046	F (1,35) = 0.002	0.966	0.966
iCAP 2	0.043	0.032	0.049	0.036	F (1,35) = 0.876	0.356	0.899
iCAP 3	0.024	0.056	0.029	0.061	F (1,35) = 0.139	0.711	0.899
iCAP 4	0.024	0.061	0.014	0.062	F (1,35) = 12.659	0.001	0.014
iCAP 5	0.009	0.038	0.008	0.047	F (1,35) = 0.08	0.779	0.899
iCAP 6	0.063	0.081	0.024	0.040	F (1,35) = 0.1	0.754	0.899
iCAP 7	0.395	0.124	0.423	0.098	F (1,35) = 0.185	0.670	0.899
iCAP 8	0.024	0.045	0.009	0.055	F (1,35) = 0.047	0.830	0.899
iCAP 9	-0.393	0.118	-0.390	0.110	F (1,35) = 0.649	0.426	0.899
iCAP 10	0.041	0.085	0.026	0.078	F (1,35) = 0.507	0.481	0.899
iCAP 11	0.029	0.047	0.040	0.049	F (1,35) = 0.124	0.727	0.899
iCAP 12	0.019	0.048	0.064	0.039	F (1,35) = 2.479	0.124	0.808
iCAP 13	0.030	0.076	0.022	0.090	F (1,35) = 1.035	0.316	0.899

Table S7. *Associations between socio-emotional outcomes and occurrence of the 13 iCAPs in the very preterm (VPT) and full-term (FT) groups based on the PLSC analysis, corresponding to Figure 1. The table shows mean saliences, bootstrap-estimated standard deviations and bootstrap ratio Z-scores for socio-emotional and iCAPs occurrence measures for the significant latent component 1.*

**Salience type: Socio-emotional measures**	**Very preterm group**		**Full-term control group**	
	Mean salience (bootstrap estimated standard deviation)	Bootstrap ratio Z-scores	Mean salience (bootstrap estimated standard deviation)	Bootstrap ratio Z-scores
Affect recognition	*0.05 (0.11)*	*0.80*	*0.26 (0.12)*	*4.97*
Theory of mind	*-0.11 (0.15)*	*-0.82*	*0.14 (0.13)*	*4.42*
Internalised problems	*-0.03 (0.15)*	*-0.47*	*-0.02 (0.12)*	*-2.10*
Emotional control	*0.35 (0.17)*	*2.47*	*0.09 (0.15)*	*-1.65*

**Coupling duration measures**	**For both the very preterm and full-term groups**	
	Mean salience (bootstrap estimated standard deviation)	Bootstrap ratio Z-scores
iCAP 1	*0.02 (0.1)*	*-1.55*
iCAP 2	*-0.13 (0.14)*	*-1.37*
iCAP 3	*-0.2 (0.11)*	*-3.74*
iCAP 4	*-0.18 (0.12)*	*-2.46*
iCAP 5	*-0.01 (0.12)*	*-1.61*
iCAP 6	*-0.07 (0.11)*	*-3.20*
iCAP 7	*-0.1 (0.1)*	*-1.71*
iCAP 8	*-0.07 (0.12)*	*-2.56*
iCAP 9	*-0.14 (0.1)*	*-3.54*
iCAP 10	*0.01 (0.1)*	*-3.28*
iCAP 11	*0.2 (0.11)*	*0.01*
iCAP 12	*-0.14 (0.14)*	*-2.02*
iCAP 13	*0.12 (0.12)*	*2.33*

Table S8*. Associations between socio-emotional outcomes and coupling duration of the 13 pair of iCAPs in the very preterm (VPT) and full-term (FT) groups based on the PLSC analysis, corresponding to Figure 2. The table shows mean saliences, bootstrap-estimated standard deviations and bootstrap ratio Z-scores for socio-emotional and iCAPs coupling measures for the significant latent component 1.*

Salience type: Socio-emotional measures	***Very preterm group***		***Full-term control group***	
	Mean salience (bootstrap estimated standard deviation)	Bootstrap ratio Z-scores	Mean salience (bootstrap estimated standard deviation)	Bootstrap ratio Z-scores
Affect recognition	*-0.15 (0.07)*	*-2.63*	*-0.18 (0.07)*	*-2.55*
Theory of mind	*0.07 (0.05)*	*1.48*	*0.01 (0.08)*	*-1.14*
Internalised problems	*-0.3 (0.06)*	*-5.55*	*-0.08 (0.07)*	*-1.12*
Emotional control	*-0.82 (0.06)*	*-14.07*	*-0.15 (0.08)*	*1.08*
Coupling duration measures	***For both the very preterm and full-term groups***			
	Mean salience (bootstrap estimated standard deviation)	Bootstrap ratio Z-scores		
iCAP1 & iCAP2	*-1.34*	*-0.05 (0.05)*		
iCAP1 & iCAP3	*5.54*	*0.16 (0.04)*		
iCAP1 & iCAP4	*0.79*	*0.04 (0.05)*		
iCAP1 & iCAP5	*0.30*	*0.02 (0.06)*		
iCAP1 & iCAP6	*-6.17*	*-0.19 (0.04)*		
iCAP1 & iCAP7	*-0.19*	*-0.02 (0.05)*		
iCAP1 & iCAP8	*-0.96*	*-0.01 (0.05)*		
iCAP1 & iCAP9	*-1.43*	*-0.06 (0.04)*		
iCAP1 & iCAP10	*0.52*	*0.02 (0.05)*		
iCAP1 & iCAP11	*-1.02*	*-0.05 (0.08)*		
iCAP1 & iCAP12	*-0.72*	*-0.04 (0.05)*		
iCAP1 & iCAP13	*-2.20*	*-0.07 (0.04)*		
iCAP2 & iCAP3	*-3.12*	*-0.12 (0.05)*		
iCAP2 & iCAP4	*0.23*	*0.02 (0.04)*		
iCAP2 & iCAP5	*0.27*	*0.03 (0.04)*		
iCAP2 & iCAP6	*-0.69*	*-0.04 (0.06)*		
iCAP2 & iCAP7	*-0.32*	*-0.02 (0.06)*		
iCAP2 & iCAP8	*-2.15*	*-0.11 (0.06)*		
iCAP2 & iCAP9	*-4.16*	*-0.16 (0.04)*		
iCAP2 & iCAP10	*0.49*	*0.01 (0.05)*		
iCAP2 & iCAP11	*-1.22*	*-0.08 (0.07)*		

iCAP2 & iCAP12	*-3.02*	*-0.15 (0.05)*
iCAP2 & iCAP13	*-3.21*	*-0.11 (0.04)*
iCAP3 & iCAP4	*-1.06*	*-0.04 (0.06)*
iCAP3 & iCAP5	*-2.42*	*-0.1 (0.05)*
iCAP3 & iCAP6	*-1.15*	*-0.05 (0.07)*
iCAP3 & iCAP7	*1.63*	*0.07 (0.05)*
iCAP3 & iCAP8	*-1.92*	*-0.08 (0.05)*
iCAP3 & iCAP9	*1.65*	*0.07 (0.06)*
iCAP3 & iCAP10	*-0.98*	*-0.04 (0.07)*
iCAP3 & iCAP11	*-0.36*	*0.01 (0.07)*
iCAP3 & iCAP12	*-2.72*	*-0.13 (0.05)*
iCAP3 & iCAP13	*-2.98*	*-0.12 (0.04)*
iCAP4 & iCAP5	*-1.44*	*-0.05 (0.06)*
iCAP4 & iCAP6	*4.98*	*0.19 (0.04)*
iCAP4 & iCAP7	*-0.52*	*-0.01 (0.06)*
iCAP4 & iCAP8	*-1.18*	*-0.06 (0.05)*
iCAP4 & iCAP9	*0.06*	*0 (0.04)*
iCAP4 & iCAP10	*-3.83*	*-0.17 (0.06)*
iCAP4 & iCAP11	*-0.05*	*-0.01 (0.06)*
iCAP4 & iCAP12	*-2.07*	*-0.09 (0.06)*
iCAP4 & iCAP13	*-2.10*	*-0.08 (0.04)*
iCAP5 & iCAP6	*1.75*	*0.07 (0.06)*
iCAP5 & iCAP7	*-0.09*	*-0.03 (0.07)*
iCAP5 & iCAP8	*-9.46*	*-0.21 (0.03)*
iCAP5 & iCAP9	*-2.59*	*-0.15 (0.06)*
iCAP5 & iCAP10	*-3.98*	*-0.14 (0.05)*
iCAP5 & iCAP11	*-0.01*	*0.01 (0.05)*
iCAP5 & iCAP12	*0.32*	*0.02 (0.05)*
iCAP5 & iCAP13	*-2.36*	*-0.09 (0.05)*
iCAP6 & iCAP7	*-0.14*	*-0.02 (0.08)*
iCAP6 & iCAP8	*-1.61*	*-0.06 (0.06)*
iCAP6 & iCAP9	*-4.83*	*-0.21 (0.05)*
iCAP6 & iCAP10	*0.43*	*0.02 (0.07)*
iCAP6 & iCAP11	*-0.72*	*-0.04 (0.06)*
iCAP6 & iCAP12	*-0.34*	*-0.01 (0.04)*
iCAP6 & iCAP13	*-3.51*	*-0.15 (0.05)*
iCAP7 & iCAP8	*0.04*	*0.01 (0.07)*

iCAP7 & iCAP9	*-1.67*	*-0.08 (0.05)*
iCAP7 & iCAP10	*-0.09*	*0 (0.06)*
iCAP7 & iCAP11	*-1.88*	*-0.07 (0.06)*
iCAP7 & iCAP12	*-2.84*	*-0.12 (0.06)*
iCAP7 & iCAP13	*0.19*	*0 (0.05)*
iCAP8 & iCAP9	*-1.71*	*-0.07 (0.06)*
iCAP8 & iCAP10	*-3.07*	*-0.12 (0.05)*
iCAP8 & iCAP11	*-0.20*	*0.01 (0.06)*
iCAP8 & iCAP12	*3.50*	*0.13 (0.04)*
iCAP8 & iCAP13	*-2.21*	*-0.1 (0.06)*
iCAP9 & iCAP10	*4.55*	*0.14 (0.04)*
iCAP9 & iCAP11	*-2.64*	*-0.09 (0.05)*
iCAP9 & iCAP12	*-3.24*	*-0.12 (0.04)*
iCAP9 & iCAP13	*-4.14*	*-0.12 (0.05)*
iCAP10 & iCAP11	*-1.77*	*-0.07 (0.05)*
iCAP10 & iCAP12	*-1.07*	*-0.05 (0.06)*
iCAP10 & iCAP13	*-0.67*	*-0.03 (0.04)*
iCAP11 & iCAP12	*-2.41*	*-0.09 (0.04)*
iCAP11 & iCAP13	*-4.10*	*-0.13 (0.04)*
iCAP12 & iCAP13	*-0.56*	*-0.02 (0.07)*
